# Effects of Strength Training on the Physiological Determinants of Middle- and Long-Distance Running Performance: A Systematic Review

**DOI:** 10.1007/s40279-017-0835-7

**Published:** 2017-12-16

**Authors:** Richard C. Blagrove, Glyn Howatson, Philip R. Hayes

**Affiliations:** 1 Faculty of Health, Education and Life Sciences, School of Health Sciences, Birmingham City University, City South Campus, Westbourne Road, Edgbaston, Birmingham, B15 3TN UK; 2 Division of Sport, Exercise and Rehabilitation, Faculty of Health and Life Sciences, Northumbria University, Northumberland Building, Newcastle-upon-Tyne, NE1 8ST UK; 3 Water Research Group, Northwest University, Potchefstroom, South Africa

## Abstract

**Background:**

Middle- and long-distance running performance is constrained by several important aerobic and anaerobic parameters. The efficacy of strength training (ST) for distance runners has received considerable attention in the literature. However, to date, the results of these studies have not been fully synthesized in a review on the topic.

**Objectives:**

This systematic review aimed to provide a comprehensive critical commentary on the current literature that has examined the effects of ST modalities on the physiological determinants and performance of middle- and long-distance runners, and offer recommendations for best practice.

**Methods:**

Electronic databases were searched using a variety of key words relating to ST exercise and distance running. This search was supplemented with citation tracking. To be eligible for inclusion, a study was required to meet the following criteria: participants were middle- or long-distance runners with ≥ 6 months experience, a ST intervention (heavy resistance training, explosive resistance training, or plyometric training) lasting ≥ 4 weeks was applied, a running only control group was used, data on one or more physiological variables was reported. Two independent assessors deemed that 24 studies fully met the criteria for inclusion. Methodological rigor was assessed for each study using the PEDro scale.

**Results:**

PEDro scores revealed internal validity of 4, 5, or 6 for the studies reviewed. Running economy (RE) was measured in 20 of the studies and generally showed improvements (2–8%) compared to a control group, although this was not always the case. Time trial (TT) performance (1.5–10 km) and anaerobic speed qualities also tended to improve following ST. Other parameters [maximal oxygen uptake ($\dot{V}{\text{O}}_{{2{ \hbox{max} }}}$), velocity at $\dot{V}{\text{O}}_{{2{ \hbox{max} }}}$, blood lactate, body composition] were typically unaffected by ST.

**Conclusion:**

Whilst there was good evidence that ST improves RE, TT, and sprint performance, this was not a consistent finding across all works that were reviewed. Several important methodological differences and limitations are highlighted, which may explain the discrepancies in findings and should be considered in future investigations in this area. Importantly for the distance runner, measures relating to body composition are not negatively impacted by a ST intervention. The addition of two to three ST sessions per week, which include a variety of ST modalities are likely to provide benefits to the performance of middle- and long-distance runners.

## Key Points

**Table:** 

Strength training (ST) appears to provide benefits to running economy, time trial performance and maximal sprint speed in middle- and long-distance runners of all abilities
Maximal oxygen uptake, blood lactate parameters, and body composition appear to be unaffected by the addition of ST to a distance runner’s program
Adding ST, in the form of heavy resistance training, explosive resistance training, and plyometric training performed, on 2–3 occasions per week is likely to positively affect performance.

## Introduction

Distance running performance is the consequence of a complex interaction of physiological, biomechanical, psychological, environmental, and tactical factors. From a physiological perspective, the classic model [1, 2] identifies three main parameters that largely influence performance: maximal oxygen uptake ($\dot{V}{\text{O}}_{{2{ \hbox{max} }}}$), running economy (RE), and fractional utilization (sustainable percentage of $\dot{V}{\text{O}}_{{2{ \hbox{max} }}}$). Collectively, these determinants are capable of predicting 16 km performance with more than 95% accuracy in well-trained runners [3]. The velocity associated with $\dot{V}{\text{O}}_{{2{ \hbox{max} }}}$ (${\text{v}}\dot{V}{\text{O}}_{{2{ \hbox{max} }}}$) also provides a composite measure of $\dot{V}{\text{O}}_{{2{ \hbox{max} }}}$ and RE, and has been used to explain differences in performance amongst trained distance runners [3, 4]. Whilst $\dot{V}{\text{O}}_{{2{ \hbox{max} }}}$ values differ little in homogenous groups of distance runners, RE displays a high degree of interindividual variability [5, 6]. Defined as the oxygen or energy cost of sustaining a given sub-maximal running velocity, RE is underpinned by a variety of anthropometric, physiological, biomechanical, and neuromuscular factors [7]. Traditionally, chronic periods of running training have been used to enhance RE [8, 9]; however, novel approaches such as strength training (ST) modalities have also been shown to elicit improvements [10].

For middle-distance (800–3000 m) runners, cardiovascular-related parameters associated with aerobic energy production can explain a large proportion of the variance in performance [11–17]. However a large contribution is also derived from anaerobic sources of energy [14, 18]. Anaerobic capabilities can explain differences in physiological profiles between middle- and longer-distance runners [14] and are more sensitive to discriminating performance in groups of elite middle-distance runners than traditional aerobic parameters [19]. Anaerobic capacity and event-specific muscular power factors, such as v$\dot{V}{\text{O}}_{{2{ \hbox{max} }}}$ and the velocity achieved during a maximal anaerobic running test (vMART) have also been proposed as limiting factors for distance runners [12, 20, 21]. For an 800-m runner in particular, near-maximal velocities of running are reached during the first 200 m of the race [22], which necessitate a high capacity of the neuromuscular and anaerobic system.

Both RE and anaerobic factors, (i.e., speed, anaerobic capacity and vMART) rely on the generation of rapid force during ground contact when running [23, 24]. Programs of ST provide an overload to the neuromuscular system, which improves motor unit recruitment, firing frequency, musculotendinous stiffness, and intramuscular co-ordination, and therefore potentially provides distance runners with a strategy to enhance their RE and event-specific muscular power factors [19]. In addition, an improvement in force-generating capacity would theoretically allow athletes to sustain a lower percentage of maximal strength, thereby reducing anaerobic energy contribution [25]. This reduction in relative effort may therefore reduce RE and blood lactate (BL) concentration. As v$\dot{V}{\text{O}}_{{2{ \hbox{max} }}}$is a function of RE, $\dot{V}{\text{O}}_{{2{ \hbox{max} }}}$ and anaerobic power factors, it would also be expected to show improvements following an ST intervention. Several recent reviews in this area have provided compelling evidence that a short-term ST intervention is likely to enhance RE [10, 26], in the order of ~ 4% [10]. Whilst these reviews have provided valuable insight into how ST specifically impacts RE, studies also typically measure other important aerobic and anaerobic determinants of distance running performance, which have not previously been fully synthesized in a review. Body composition also appears to be an important determinant of distance running performance, with low body mass conferring an advantage [27, 28]. Resistance training (RT) is generally associated with a hypertrophic response [29]; however, this is known to be attenuated when RT and endurance training are performed concurrently within the same program [30]. Changes in body composition as a consequence of ST in distance runners have yet to be fully addressed in reviews on this topic.

There are also a number of recent publications [31–38] that have not been captured in previous reviews [10, 26] on this topic, which potentially provide valuable additional insight into the area. Previous papers that have reviewed the impact of ST modalities on distance running performance have done so alongside other endurance sports [23, 39] or are somewhat outdated [40–42]. Furthermore, although improvements in RE would likely confer a benefit to distance running performance, the outcomes from studies that have used time trials have not been comprehensively reviewed. Performance-related outcome measures provide high levels of external validity compared to physiological parameters, therefore it is likely that a collective summary of results would be of considerable interest to coaches and athletes.

Consequently the aim of this review was to systematically analyze the evidence surrounding the use of ST on distance running parameters that includes both aerobic and anaerobic qualities, in addition to body composition and performance-related outcomes. This work also provides a forensic, critical evaluation that, unlike previous work, highlights areas that future investigations should address to improve methodological rigor, such as ensuring valid measurement of physiological parameters and maximizing control over potential confounding factors.

## Methods

### Literature Search Strategy

The PRISMA statement [43] was used as a basis for the procedures described herein. Electronic database searches were carried out in Pubmed, SPORTDiscus, and Web of Science using the following search terms and Boolean operators: (“strength training” OR “resistance training” OR “weight training” OR “weight lifting” OR “plyometric training” OR “concurrent training”) AND (“distance running” OR “endurance running” OR “distance runners” OR “endurance runners” OR “middle distance runners”) AND (“anaerobic” OR “sprint” OR “speed” OR “performance” OR “time” OR “economy” OR “energy cost” OR “lactate” OR “maximal oxygen uptake” OR “$\dot{V}{\text{O}}_{{2{ \hbox{max} }}}$” OR “aerobic” OR “time trial”). Searches were limited to papers published in English and from 1 January 1980 to 6 October 2017.

### Inclusion and Exclusion Criteria

For a study to be eligible, each of the following inclusion criteria were met:

- Participants were middle- (800–3000 m) or long-distance runners (5000 m–ultra-distance). Studies using triathletes and duathletes were also included because often these participants possess similar physiology to distance runners and complete similar volumes of running training.
- A ST intervention was applied. This was defined as heavy (less than 9 repetition maximum (RM) loads and/or 80% of 1RM) or isometric resistance training (HRT), moderate load (9–15 RM and/or 60–80% 1RM) RT, explosive resistance training (ERT), reactive ST or plyometric training (PT). Sprint training (SpT) could be used in conjunction with one or more of the above ST methods, but not exclusively as the only intervention activity.
- The intervention period lasted 4 weeks or longer. This criteria was employed as neuromuscular adaptations have been observed in as little as 4 weeks in non-strength trained individuals [44, 45].
- A running only control group was used that adopted similar running training to the intervention group(s).
- Data on one or more of the following physiological parameters was reported: $\dot{V}{\text{O}}_{{2{ \hbox{max} }}}$, RE, velocity associated with v$\dot{V}{\text{O}}_{{2{ \hbox{max} }}}$, time trial (TT) performance, time to exhaustion (TTE), BL response, anaerobic capacity, maximal speed, measures of body composition.
- Published in full in a peer-reviewed journal.

Studies were excluded if any of the following criteria applied:

- Participants were non-runners (e.g., students, untrained/less than 6 months running experience). Further restrictions were not placed upon experience/training status.
- The running training and/or ST intervention was poorly controlled and/or reported.
- The intervention involved only SpT or was embedded as part of running training sessions.
- Participants were reported to be in poor health or symptomatic.
- Ergogenic aids were used as part of the intervention.

Using the mean $\dot{V}{\text{O}}_{{2{ \hbox{max} }}}$ values provided within each study, participants training status was considered as moderately-trained (male $\dot{V}{\text{O}}_{{2{ \hbox{max} }}}$ ≤ 55 ml kg^−1^ min^−1^), well-trained (male $\dot{V}{\text{O}}_{{2{ \hbox{max} }}}$ 55–65 ml kg^−1^ min^−1^), or highly-trained (male $\dot{V}{\text{O}}_{{2{ \hbox{max} }}}$ ≥ 65 ml kg^−1^ min^−1^) [10, 46]. For female participants, the $\dot{V}{\text{O}}_{{2{ \hbox{max} }}}$ thresholds were set 10 ml kg^−1^ min^−1^ lower [46]). In the absence of $\dot{V}{\text{O}}_{{2{ \hbox{max} }}}$ values, training status was based upon the training or competitive level of the participants: moderately-trained = recreational or local club, well-trained = Collegiate or provincial, highly-trained = national or international.

### Study Selection

**Fig. 1:**
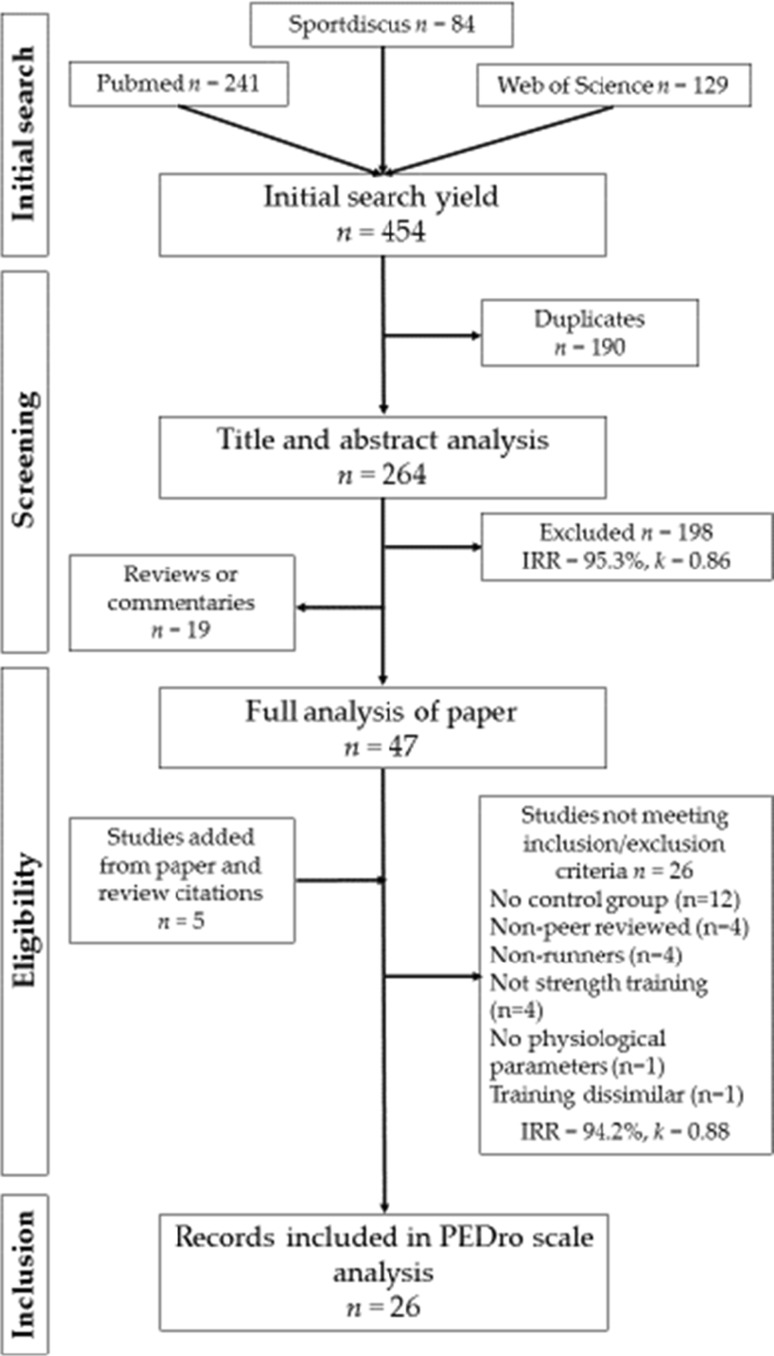
Search, screening and selection process for suitable studies

Figure 1 provides a visual overview of the study selection process. Search results were imported into a published software for systematic reviews [47], which allowed a blind screening process to be performed by two independent reviewers (RB and PH). Any disagreements were resolved by consensus. The initial search yielded 454 publications. Following the removal of duplicates (*n* = 190), publications were filtered by reading the title and abstract [inter-rater reliability (IRR): 95.3%, Cohens *k* = 0.86] leaving 19 review articles or commentaries, and 47 potentially relevant papers, which were given full consideration. Five additional records were identified as being potentially relevant via manual searches of previously published reviews on this topic and the individual study citations. These 52 studies were considered in detail for appropriateness, resulting in a further 26 papers [34, 37, 48–71] being excluded (IRR: 94.2%, Cohens *k* = 0.88) for the following reasons: not published in full in a peer-reviewed journal [50, 52, 60, 61], absence of a running only control group [48, 49, 54, 57, 59, 62–67, 69], participants were non-runners [51, 53, 56, 68], no physiological parameters were measured [55], dissimilar running training was applied between groups [71], the ST intervention was poorly controlled [54], and ST did not involve one of the aforementioned types [34, 37, 58, 70].

### Analysis of Results

The Physiotherapy Evidence Database (PEDro) scale was subsequently used to assess the quality of the remaining 26 records [31–33, 36, 38, 72–92] by the two independent reviewers. Two studies reported their results across two papers [32, 38, 90, 92], therefore both are considered as single studies hereafter, thus a total of 24 studies were analyzed. The PEDro scale is a tool recommended for assessing the quality of evidence when systematically reviewing randomized-controlled trials [93]. Each paper is scrutinized against 11 items relating to the scientific rigor of the methodology, with items 2–11 being scored 0 or 1. Papers are therefore awarded a rating from 0 to 10 depending upon the number of items which the study methodology satisfies (10 = study possesses excellent internal validity, 0 = study has poor internal validity). No studies were not excluded based upon their PEDro scale score and IRR was excellent (93.2%, Cohens *k* = 0.86).

Results are summarized as a percentage change and the *p* value for variables relating to: strength outcomes, RE, $\dot{V}{\text{O}}_{{2{ \hbox{max} }}}$, v$\dot{V}{\text{O}}_{{2{ \hbox{max} }}}$, BL response, time trial, anaerobic performance, and body composition. Due to the heterogeneity of outcome measures in the included studies and the limitations associated with conditional probability, where possible, an effect size (ES) statistic (Cohens *d*) is also provided. Effect size values are based upon those reported in the studies or were calculated using the ratio between the change score (post-intervention value minus pre-intervention value) and a pooled standard deviation at baseline for intervention and control groups. Values are interpreted as trivial < 0.2; small 0.2–0.6; moderate 0.6–1.2; and large > 1.2.

## Results

### Participant Characteristics

A summary of the participant characteristics for the 24 studies which met the criteria for inclusion in this review is presented in Table 1. A total of 469 participants (male *n* = 352, female *n* = 96) are included, aged between 17.3 and 44.8 years. Maximal oxygen uptake data was reported for all but five studies [83, 84, 86, 87, 90, 92] and ranged from 47.0 to 70.4 mL kg^−1^ min^−1^. Based upon weighted mean values in the studies that reported participant characteristics for each group, age (30.2 vs. 29.0 years), body mass (68.1 vs. 70.0 kg), height (1.74 vs. 1.74 m), and $\dot{V}{\text{O}}_{{2{ \hbox{max} }}}$ (57.3 vs. 57.7 mL kg^−1^ min^−1^) appeared to differ little at baseline for ST groups and control groups respectively. Moderately trained or recreational level runners were used in nine studies [31, 72, 76, 81, 83, 84, 86, 90–92], well-trained participants in ten studies [32, 33, 36, 38, 73, 75, 79, 80, 85, 88, 89], and highly-trained or national/international runners were used in four studies [74, 77, 82, 87]. National caliber junior runners were also used in one investigation [78]. Participants took part or competed in events ranging from the middle-distances to ultra-marathons, and several studies used triathletes [31, 74, 83] or duathletes [32, 38].

**Table 1:** Participant characteristics and design of each study

Study	Participant characteristics					Study design				
	*n* (I/C)	Sex	Age (years)	$\dot{V}{\text{O}}_{{2{ \hbox{max} }}}$ (mL kg^−1^ min^−1^)	Training background (event specialism)	Duration (weeks)	Randomized?	Running controlled?	ST added or replace running?	PEDro score
Albracht & Arampatzis [84]	26 (13/13)	*M*	*I* = 27, *C* = 25	–	Recreational (≥ 3 runs wk^−1^, 30–120 km wk^−1^)	14	No	No	Added	5
Beattie et al. [33]	20 (11/9)	*M* = 19 *F* = 1	*I* = 29.5, *C* = 27.4	*I* = 59.6, *C* = 63.2	Collegiate and national level (1500 m–10 km)	40	No	No	Added	4
Berryman et al. [80]	28 (HRT *n* = 12, PT *n* = 11, C *n* = 5)	*M*	HRT = 31, PT = 29, *C* = 29	HRT = 57.5, PT = 57.5, C = 55.7	3–7 runs wk^−1^. Provincial level (5 km–marathon)	8	Yes	Yes	Added	5
Bertuzzi et al. [85]	22 (RT_WBV_ *n* = 8, RT *n* = 8, C *n* = 6)	*M*	RT_WBV_ = 34, RT = 31, *C* = 33	RT_WBV_ = 56.3, RT = 57.4, *C* = 56.1	Local 10 km (35–45 min) race competitors	6	Yes	No (monitored)	Added	6
Bonacci et al. [83]	8 (3/5)	*M* = 5 *F* = 3	21.6	–	Moderately-trained triathletes (34.8 km wk^−1^)	8	Yes	No (monitored)	Added	5
Damasceno et al. [89]	18 (9/9)	*M*	*I* = 34.1, *C* = 32.9	*I* = 54.3, *C* = 55.8	Local 10 km (35–45 min) race competitors	8	Yes	No (monitored)	Added	6
Ferrauti et al. [81]	20 (11/9)	*M* = 14 *F* = 6	40.0	*I* = 52.0, *C* = 51.1	Experienced (8.7 years) recreational (4.6 h wk^−1^)	8	Yes	No (monitored)	Added	6
Fletcher et al. [82]	12 (6/6)	*M*	*I* = 22.2, *C* = 26.3	*I* = 67.3, *C* = 67.6	Regional/national/international level (1500 m– marathon)	8	Yes	No	Added	6
Giovanelli et al. [36]	25 (13/12)	*M*	*I* = 36.3, *C* = 40.3	*I* = 55.2, *C* = 55.6	Experienced (11.7 years, > 60 km wk^−1^) ultra-distance competitors	12	Yes	No (monitored)	Added	6
Johnston et al. [72]	12 (6/6)	*F*	30.3	*I* = 50.5, *C* = 51.5	>1 year experience, 20–30 miles wk^−1^, 4–5 days wk^−1^	10	Yes	No (monitored)	Added	6
Karsten et al. [31]	16 (8/8)	*M* = 11*F* = 5	*I* = 39, *C* = 30	*I* = 47.3, *C* = 47.0	Recreational triathletes (> 2 years, 3–5 days wk^−1^, 180–300 min wk^−1^)	6	Yes	No	Added	6
Mikkola et al. [78]	25 (13/12)	*M* = 18 *F* = 7	*I* = 17.3, *C* = 17.3	*I* = 62.4, *C* = 61.8	High-school runners (> 2 years)	8	No	No (monitored)	Replace (I: 19%, C: 4%)	4
Millet et al. [74]	15 (7/8)	*M*	*I* = 24.3, *C* = 21.4	*I* = 69.7, *C* = 67.6	Experienced (6.8 years) triathletes (*n* = 7 national/international)	14	Yes	No (monitored)	Added	6
Paavolainen et al. [73]	18 (10/8)	*M*	*I* = 23, *C* = 24	*I* = 63.7, *C* = 65.1	Experienced (8 years) cross-country runners (545 h year^−1^)	9	Unclear (matched on $\dot{V}{\text{O}}_{{2{ \hbox{max} }}}$ and 5 km)	Yes	Replace (I: 32%, C: 3%)	4
Pellegrino et al. [91]	22 (11/11)	*M* = 14 *F* = 8	*I* = 34.2, *C* = 32.5	*I* = 48.0, *C* = 47.7	Experienced recreational (local clubs and races)	6	Yes	No	Added	6
Piacentini et al. [86]	16 (HRT *n* = 6, RT *n* = 5, C *n* = 5)	*M* = 6 *F* = 4	HRT = 44.2 RT = 44.8 C = 43.2	–	Local (> 5 years, 4–5 days wk^−1^) masters runners (10 km – marathon)	6	Yes	No	Added	4
Ramírez-Campillo et al. [87]	32 (17/15)	*M* = 9 *F* = 13	22.1	–	National/international competitive level (1500 m – marathon)	6	Yes	No (monitored)	Added	6
Saunders et al. [77]	15 (7/8)	*M*	*I* = 23.4, *C* = 24.9	*I* = 67.7, *C* = 70.4	National/international competitive level (3 km)	9	Yes	No (monitored)	Added (but C matched with stretching/CS)	6
Schumann et al. [90, 92]	27 (13/14)	M	33	–	Recreational (> 12 months; ≥ 2 runs wk^−1^)	24	Unclear (matched by performance)	Yes	Added	5
Skovgaard et al. [88]	21 (12/9)	*M*	31.1	59.4	Experienced (7.5 years) recreational (29.7 km wk^−1^, 3.3 runs wk^−1^)	8	Yes	Yes (I only)	Replace (I: 42%)	6
Spurrs et al. [75]	17 (8/9)	*M*	25	*I* = 57.6, *C* = 57.8	Experienced (10 years); 60–80 km wk^−1^	6	Yes	No (monitored)	Added	6
Støren et al. [79]	17 (8/9)	*M* = 9 *F* = 8	*I* = 28.6, *C* = 29.7	*I* = 61.4, *C* = 56.5	Well-trained (5 km: *M* = 18.42, *F* = 19.23)	8	Yes	No (monitored)	Added	6
Turner et al. [76]	18 (10/8)	*M* = 8 *F* = 10	*I* = 31, *C* = 27	*I* = 50.4, *C* = 54.0	Basic training (> 6 months; ≥ 3 runs wk^−1^)	6	Yes	No (monitored)	Added	6
Vikmoen et al. [32, 38]	19 (11/8)	*F*	*I* = 31.5, *C* = 34.9	53.3	Well-trained (duathletes)	11	Yes	Yes	Added	5

*C* control group, *CS* core stability, *F* female, *h* hours, *HRT* heavy resistance training, *I* intervention group, *M* male, *PT* plyometric training, *RT* resistance training, *RT*_*WBV*_ resistance training with whole body vibration, $\dot{V}{\text{O}}_{{2{ \hbox{max} }}}$ maximal oxygen uptake, *wk* week

### Study Design and PEDro Scores

Table 1 also provides an overview of several important features of study design, including PEDro scale scores. Studies lasted 6–14 weeks with the exception of two investigations, which lasted 24 [90, 92] and 40 weeks [33]. Fourteen studies provided detailed accounts of the running training undertaken by the participants. However, these were usually reported from monitoring records, thus only three studies were deemed to have appropriately controlled for the volume and intensity of running in both groups [32, 38, 73, 80, 90, 92]. Six studies provided little or no detail on the running training that participants performed [31, 33, 82, 84, 86, 91]. Strength training in all but three investigations [73, 78, 88] was supplementary to running training, and one paper provided the control group with alternative activities (stretching and core stability) matched for training time [77].

Studies were all scored a 4, 5, or 6 on the PEDro scale. All investigations had points deducted for items relating to blinding of participants, therapists, and assessors. Differences in the scores awarded were mainly the result of studies not randomly allocating participants to groups and failing to obtain data for more than 85% of participants initially allocated to groups; or this information not being explicitly stated.

### Training Programs

Table 2 provides a summary of the training characteristics associated with the ST intervention and running training used concurrently as part of the study period. The ST activities used were RT or HRT [31, 32, 38, 72, 78, 79, 81, 82, 84–86, 89], PT [75, 76, 80, 87, 91], ERT [80], or a combination of these methods [33, 36, 77, 83, 90, 92], which in some cases also included SpT [73, 74, 88].

**Table 2:** Intervention and running training variables

Study	Intervention type	Main exercises	Frequency	Volume per session	Intensity	ST supervised?	Recovery between sessions	Running training
Albracht & Arampatzis [84]	HRT (isometric)	Ankle plantarflexion (5^°^ dorsiflexion, knee extended, 40^°^ hip flexion)	4 per week	4 sets × 4 reps (3 s loading, 3 s relaxation)	90% MVC (adjusted weekly)	Yes	–	I: 66 km wk^−1^ C: 62 km wk^−1^
Beattie et al. [33]	HRT/ERT/PT	PT: pogo jumps, depth jumps, CMJ HRT: back squat, RDL, lunge ERT: jump squats	Wk 1–20: 2 per week; Wk 21–40: 1 per week	9–12 sets (2–3 sets per exercise); PT: 4–5 reps, HRT: 3–8 reps, ERT: 3 reps	Load progressed when competent	Yes	≥48 h between sessions (wk 1–20). Separate session to running	Not reported (usual running training)
Berryman et al. [80]	ERT and PT	ERT: concentric squats PT: DJ	1 per week	ERT and PT: 3–6 sets × 8 reps	ERT: > 95% PPO PT: 20–60 cm so rebound > 95% CMJ	Yes	–	2 × AIT (1 × peak speed, 1 × 80% peak speed) 1 × LSD (30–60 min)
Bertuzzi et al. [85]	RT and RT_WBV_	Half-squats	2 per week	3–6 sets × 4–10 reps periodized	70–100% 1RM over 12 wk	Yes	Different days to runs	57–61 km wk^−1^
Bonacci et al. [83]	PT/ERT	PT: CMJ, knee lifts, ankle jumps, bounds, skips, hurdle jumps ERT: Squat jumps, back ext., hamstring curls	3 per week	PT: 1–5 sets × 5–10 reps or 20–30 m RT: 2–5 sets × 8–15 reps	Max height/fast velocity	Yes	–	Same as previous 3 months. I: swim (7.3 km), cycle (137.6 km), run (34.8 km) C: swim (10.1 km), cycle (147.5 km), run (29.0 km)
Damasceno et al. [89]	HRT	Half-squat, leg press, calf raise, knee ext	2 per week	2–3 sets × 3–10 reps	10RM periodized to 3RM	Yes	72 h between HRT sessions. Different days to runs	36–41 km wk^−1^ @50–70% $\dot{V}{\text{O}}_{{2{ \hbox{max} }}}$
Ferrauti et al. [81]	HRT	Machines: leg press, knee ext., knee flexion, hip ext., ankle ext.; UB exercises	1 per week LB; 1 per week UB	LB: 4 sets × 3–5 reps	3–5 RM	Yes	–	I: 240 min wk^−1^, C: 276 min wk^−1^
Fletcher et al. [82]	HRT (isometric)	Plantarflexions	3 per week	4 sets × 20 s	80% MVC	Yes	–	70–170 km wk^−1^
Giovanelli et al. [36]	CS/RT (4wk) HRT/ERT/PT (8wk)	CS: 6 exercises (e.g., planks) RT/HRT: single leg half-squat, step-up, lunges ERT: CMJ, split squat PT: jump rope, high knees	3 per week	5–8 exercises, 1–3 sets × 6–15 reps (30 s rest)	–	Partly (only wk 1 and 2)	≥48 h between sessions. Not day after races/AIT	I: normal running training C: 70–140 km wk^−1^, 5–7 sessions wk^−1^
Johnston et al. [72]	HRT	Squats, lunge, heel raises (straight- and bent-leg), knee ext./flexion, 8xUB exercises	3 per week	3 sets × 6 reps squat and lunge; 2 sets × 20/12 reps bent–/straight–leg heel raise; 3 sets × 8 reps knee ext./flexion	RM each set	Yes	≥48 h between HRT sessions. ≥ 5 h between HRT and running sessions.	4–5 days wk^−1^, 32–48 km wk^−1^
Karsten et al. [31]	HRT	RDL, squat, calf raises, lunges	2 per week	4 sets × 4 reps	80% 1RM	Yes	≥48 h between HRT sessions.	3–5 sessions/180–300 min wk^−1^
Mikkola et al. [78]	HRT	Hamstring curl, leg press, seated press, squat, leg ext., heel raise	2 per week	3–5 sets × 3–5 reps	>90% 1RM (reassessed every 3 wk)	Yes	Separate session to running	Total: *I* = 7 h wk^−1^, *C* = 6.6 h wk^−1^; Running: *I* = 48 km wk^−1^, *C* = 44 km wk^−1^
Millet et al. [74]	SpT/PT/ERT	PT: alternative, calf, squat, hurdle jumps ERT: Squat, calf raise, hurdle jump, leg ext./curl	3 per week (each intervention type once)	SpT: 5–10 sets × 30–150 m PT/ERT: 2–3 sets × 6–10 reps	PT: BW ERT: low load, high velocity	Unclear	–	*I*: 8.8 h wk^−1^, *C*: 8.5 h wk^−1^
Paavolainen et al. [73]	SpT/PT/ERT	PT: alternative, drop and hurdle jumps, CMJ, hops ERT: leg press, knee ext. and flexion	Not reported; 2.7 h per week	SpT: 5–10 sets × 20–100 m PT/ERT: 5–20 reps.set^−1^/30–200 reps.session^−1^	PT: BW or barbell ERT: 0–40% 1RM	Unclear	–	*I*: 8.4 h wk^−1^ (9 sessions) *C*: 9.2 h wk^−1^ (8 sessions)
Pellegrino et al. [91]	PT	Modified version of Spurrs et al. (jumps, bounds, hops)	15 sessions total	60–228 foot contacts	Progressively increased	Yes	–	I: 34.4–36.2 km wk^−1^ C: 29.5–31.3 km wk^−1^
Piacentini et al. [86]	HRT and RT	Squat, calf press, lunges, eccentric quad, calf raise, leg press + UB exercises	2 per week	HRT: 4 sets × 3–4 reps RT: 3 sets × 10 reps	HRT: 85–90% 1RM RT: 70% 1RM	Yes	–	4–5 days wk^−1^, 50 km wk^−1^
Ramírez-Campillo et al. [87]	PT	DJ	2 per week	60 contacts (6 sets × 10 reps)	20 reps @20 cm, 20 reps @40 cm, 20 reps @60 cm	Yes	≥48 h between PT sessions. Performed before runs.	I: 64.7 km.wk^−1^ C: 70.0 km.wk^−1^ (AIT preferred)
Saunders et al. [77]	PT/HRT	PT: CMJ, ankle jumps, bounds, skips, hurdle jumps, scissor jumps HRT: back ext., leg press, hamstring curls	3 per week	PT: Progress from 1 to 6 sets × 6–10 reps/10–30 m HRT: 1–5 sets × 6–10 reps (except back ext.)	PT: fast GCT HRT: Leg press 60% 1RM	Yes	–	107 km.wk^−1^ (3x AIT, 1 × LSD 60–150 min, 3 × LSD 30–60 min, 3–6 × LSD 20–40 min)
Schumann et al. [90, 92]	HRT/ERT/PT	HRT: leg press, knee flexion, calf raise +UB/core exercises ERT: Squat jumps, step-ups PT: Drop jumps, hurdle jumps	2 per week	HRT (wk 5–24): 5–12 reps per set	HRT (wk 5–24): 60–85% 1RM ERT: 20–30% 1RM	Yes	Same session as running. >48 h between sessions	Weekly: 2x run (35–45 min/65–85% HR_max_), 2 × LSD (35–40 min & 70–125 min/60–65% HR_max_), 1–2 × AIT and HIIT
Skovgaard et al. [88]	SpT/HRT	HRT: squat, deadlift, leg press	SpT × 2 per week HRT × 1 per week	SpT: 4–12 sets × 30 s (3 min rest) HRT: 3–4 sets × 6–8 reps wk 1–4; 4 sets × 4 reps wk 5–8	SpT: maximal effort HRT: 15RM to 8RM wk 1–4; 4RM wk 5–8	Yes	3–4 d between SpT/HRT sessions. Different days to runs	I: AIT (4 × 4 + 2 min @85% HR_max_); 50 min @75–85% HR_max_ C: 40 km total (4 km AIT)
Spurrs et al. [75]	PT	Jumps, bounds, hops	2–3 per week	60–180 foot contacts	Bilateral progressed to unilateral and greater height	Yes	Separate session to running	60–80 km per week
Støren et al. [79]	HRT	Half-squats	3 per week	4 sets × 4 reps	4RM	Yes	–	*I*: 253 min wk^−1^ (+ 119 min other ET) *C*: 154 min wk^−1^ (+120 min other ET)
Turner et al. [76]	PT	Vertical jumps and hops (continuous and intermittent), split jumps, uphill jumps	3 per week	40–110 foot contacts (5–30 s per exercise)	Bodyweight, short contact time	No (logbooks)	Performed in running sessions	Continued regular running (≥ 3 runs wk^−1^, ≥ 10 miles wk^−1^)
Vikmoen et al. [32, 38]	HRT	Machines: Half-squats, unilateral leg press, cable hip flexion, calf raises	2 per week	3 sets × 4–10 reps (periodized 3wk cycles)	Sets performed to RM failure	Partly (1 session per wk 3–11)	HRT first session or performed on different days	4.3 sessions wk^−1^; 3.7 h @60–82% HR_max_, 1.1 h @83–87% HR_max_, 0.8 h @ > 87% HR_max_

*AIT* aerobic interval training, *BW* body weight, *CMJ* counter-movement jump, *C* control group, *CS* core stability, *DJ* drop jump, *ERT* explosive resistance training, *ET* endurance training (e.g., cycling, swimming, roller skiing), *GCT* ground contact time, *h* hours, *HIIT* high-intensity interval training, *HR*_*max*_ maximum heart rate (predicted from 220-age), *HRT* heavy resistance training, *I* intervention group, *LB* lower body, *LSD* long slow distance run, *MVC* maximum voluntary contraction, *PPO* peak power output, *PT* plyometric training, *RDL* Romanian deadlift, *RM* repetition maximum, *RT* resistance training, *SpT* sprint training, *ST* strength training, *UB* upper body, *RT*_*WBV*_ resistance training with whole body vibration

All studies utilized at least one multi-joint, closed kinetic chain exercise with the exception of two studies that used isometric contractions on the ankle plantarflexors [82, 84]. One study employed only resistance machine exercises for lower limb HRT [81], whereas all other studies used free weights, bodyweight resistance or a combination of machines and free weights. Strength training (using lower limb musculature) was scheduled once [33, 80, 81], twice [31–33, 38, 75, 78, 85–87, 89, 90, 92], three times [36, 72, 74–77, 79, 82, 83, 88], or four times [84] per week. One study used 15 sessions over a 6-week period [91] and one study reported 2.7 h of ST activity per week [73].

Heavy RT was typically prescribed in 2–6 sets of 3–10 repetitions per exercise at relatively heavy loads (higher than 70% 1RM or to repetition failure). Plyometric training prescription consisted of 1–6 exercises performed over 1–6 sets of 4–10 repetitions, totaling 30–228 foot contacts per session. Most studies applied the principle of progressive overload and some authors reported periodized models for the intervention period [32, 33, 36, 38, 77, 88, 89]. Studies which included SpT tended to utilize short distances (20–150 m), over 4–12 sets at maximal intensity [73, 74, 88]. Strength training was supervised or part-supervised across all studies with the exception of three, one that was unsupervised [76] and two where it was unclear from the report [73, 74].

Running training varied considerably (16–170 km week^−1^, 3–9 sessions week^−1^) across the studies, with various levels of detail provided regarding weekly volume and intensity. Importantly, all studies that added ST reported that running training did not differ between groups.

### Strength Outcomes

All but two studies [31, 83] measured at least one strength-related parameter (Table 3). Across all studies that used 1RM testing [33, 72, 74, 78, 79, 85, 86, 88–90, 92], the intervention produced a statistically significant improvement (4–33%, ES: 0.7–2.4). Maximal voluntary contraction (MVC) was also used to assess strength capacity in seven papers, with the majority reporting improved (7–34%, ES: 0.38–1.65) scores following ST [73, 75, 78, 81, 84] but others reporting no difference compared to a control group [81, 82, 90, 92]. Performance on a jump test was shown to improve (3–9%, ES: 0.25–0.65) in some studies [32, 73, 74, 80, 87]; however, other studies showed no change compared to a control group [33, 76–78, 90–92] and in one study the control group improved to a greater extent than the intervention group [86]. Changes in an ability to produce force rapidly also showed mixed results, with some studies showing improvements in peak power output [80] and rate of force development [78, 79] and others showing no change in these parameters [36, 75, 77]. Similarly, stiffness, when measured directly or indirectly (using reactive strength index) during non-running tasks, has been shown to improve (ES: 0.43–0.90) [75, 84, 86, 87] and remain unchanged [33, 74, 89] following ST. Vertical or leg stiffness during running showed improvements (10%, ES: 0.33) at relatively slow speeds [36] and also at 3 km race pace (ES: 1.2) following ST [74].

**Table 3:** Outcomes of the studies. Percentage changes, effect size (ES) and *p* value only reported for statistically significant group results or ES > 0.2. All results presented are for the intervention (I) group unless stated (e.g., C = control). Variables measured where no-significance (NS) difference for time (pre- vs. post-score) and no group × time (G × T) interaction was detected, are also listed

Study	Main strength outcomes	Economy	$\dot{V}{\text{O}}_{{2{ \hbox{max} }}} /\dot{V}{\text{O}}_{{2{\text{peak}}}}$	v$\dot{V}{\text{O}}_{{2{ \hbox{max} }}}$	Blood lactate	Time trial	Anaerobic measures	Body composition
Albracht and Arampatzis [84]	Plantarflexion MVC (6.7%, ES = 0.56, *p* = 0.004), max Achilles tendon force (7.0%, ES = 0.55, *p* < 0.01), Tendon stiffness (15.8%, ES = 0.90, *p* < 0.001)	$\dot{V}{\text{O}}_{2}$@10.8 km h^−1^ (5.0%, ES = 0.79) @12.6 km h^−1^ (3.4%, ES = 0.51) EC@10.8 km h^−1^ (4.6%, ES = 0.61) @12.6 km h^−1^ (3.5%, ES = 0.50), all *p* < 0.05	–	–	BL@10.8 and 12.6 km h^−1^, NS	–	–	Body mass, NS
Beattie et al. [33]	1RM back squat (wk 0–20: 19.3%, ES = 1.2, *p* = 0.001) DJ_RSI_ (wk 0–20: 7.3%, ES = 0.3, NS G × T; wk 0–40: 14.6%, ES = 0.5, NS G × T) CMJ (wk 0–20: 11.5%, ES = 0.5, NS G × T; wk 0–40: 11.5%, ES = 0.6, NS G × T)	Ave. of 5 speeds Wk 0–20: 5.0%, ES = 1.0, *p* = 0.01. Wk 0–40: 3.5%, ES = 0.6, NS.	Wk 0–20: 0.1%, ES = 0.1, *p* = 0.013. Wk 0-40, I: 7.4%, ES = 0.5, *p* = 0.003, C: 2.8%, ES = 0.6, NS	Wk 0-20: 3.5%, ES = 0.7, NS. Wk 0-40: 4.0%, ES = 0.9, NS	v2 mmol L^−1^, v4 mmol L^−1^, NS	–	–	Body mass, fat and lean muscle, NS
Berryman et al. [80]	PPO (ERT: 15.4%, ES = 0.98, *p* < 0.01; PT: 3.4%, ES = 0.24, *p* < 0.01). CMJ (ERT: 4.5%, ES = 0.25, *p* < 0.01; PT: 6.0%, ES = 0.52, *p* < 0.01)	@12 km h^−1^ ERT: 4%, ES = 0.62, *p* < 0.01. PT: 7%, ES = 1.01, *p* < 0.01	NS	ERT: 4.2%, ES = 0.43, *p* < 0.01. PT: 4.2%, ES = 0.49, *p* < 0.01	–	3 km TT ERT: 4.1%, ES = 0.37. PT: 4.8%, ES = 0.46. C: 3.0%, ES = 0.20; all *p* < 0.05, G × T NS	–	Body mass, NS
Bertuzzi et al. [85]	1RM half squat (RT: 17%, *p* ≤ 0.05; RT_WBV_: 18%, *p* ≤ 0.05)	–	NS	NS	–	–	–	–
Bonacci et al. [83]	–	@12 km h^−1^ (after 45 min AIT cycle) NS	–		–	–	–	Body mass, skinfolds, thigh and calf girth, NS
Damasceno et al. [89]	1RM half–squat (23%, ES = 1.41, *p* < 0.05), DJ_RSI_, wingate test NS	@12 km h^−1^ NS	NS	v$\dot{V}{\text{O}}_{{2{ \hbox{max} }}}$ (2.9%, ES = 0.42, *p* < 0.05)	–	10 km TT (2.5%, *p* = 0.039), increased speed in final 7 laps (*p* < 0.05)	30 s Wingate test, NS	Body mass and skinfold, NS
Ferrauti et al. [81]	Leg extension MVC (33.9%, ES = 1.65, *p* < 0.001); leg flexion MVC (9.4%, ES = 0.38, NS)	@LT (ES = 0.40, *p* < 0.05, NS G × T) @8.6 and 10.1 km h^−1^, NS FU@10.1 km h^−1^ (ES = 0.61, *p* = 0.05 G × T)	5.6%, ES = 0.40, NS G × T	–	BL@10.1 km h^−1^ (I: 13.1%, C: 12.1%, NS G × T). v4 mmol L^−1^ (I: 4.2%, C: 2.6%, NS G × T).	–	–	Body mass, NS
Fletcher et al. [82]	Isometric MVC (I: 21.6%, C: 13.4%), NS G × T	EC@75,85,95% sLT, NS	–	–	BL@ 75,85,95% sLT, NS.	–	–	–
Giovanelli et al. [36]	SJ PPO, NS *k*_leg_@10 km h^−1^, (9.5%, ES = 0.33, *p* = 0.034), @12 km h^−1^ (10.1%, ES = 0.33, *p* = 0.038). *k*_vert_ @8,10,12,14 km h^−1^, NS	@8 km h^−1^ (6.5%, ES = 0.43, *p* = 0.005), @10 km h^−1^ (3.5%, ES = 0.48, *p* = 0.032), @12 km h^−1^ (4.0%, ES = 0.34, *p* = 0.020), @14 km h^−1^ (3.2%, ES = 0.35, *p* = 0.022), @RCP NS	NS	NS	–	–	–	Body mass, FFM, fat mass, NS
Johnston et al. [72]	1RM squat (40%, *p* < 0.05), knee flexion (27%, *p* < 0.05)	@12.8 km h^−1^ (4.1%, ES = 1.76, *p* < 0.05), @13.8 km h^−1^ (3.8%, ES = 1.61, *p* < 0.05)	NS	–	–	–	–	Body mass, fat mass, FFM, limb girth, NS
Karsten et al. [31]	–	–	NS	NS	–	5 km TT (3.5%, ES = 1.06, *p* = 0.002)	ARD, NS	–
Mikkola et al. [78]	MVC (8%), 1RM (4%), RFD (31%) on leg press; all *p* < 0.05. CMJ and 5–bounds, NS	@14 km h^−1^ (2.7%, ES = 0.32, p < 0.05), @10,12,13 km h^−1^, NS	NS	NS	BL@12 km h^−1^ (12%, p < 0.05), @14 km h^−1^ (11%, *p* < 0.05)	–	vMART (3.0%, *p* < 0.01), v30 m sprint (1.1%, *p* < 0.01)	Body mass (2%, ES = 0.32, *p* < 0.01). Thickness of QF (I: 3.9%, ES = 0.35, *p* < 0.01; C: 1.9%, ES = 0.10, *p* < 0.05); fat %, lean mass, NS
Millet et al. [74]	1RM half–squat (25%, *p* < 0.01), 1RM heel raise (17%, *p* < 0.01), hop height (3.3%, *p* < 0.05) *k*_leg_@3 km pace (ES = 1.2, *p* < 0.05) GCT, hop stiffness, NS	@75% v$\dot{V}{\text{O}}_{{2{ \hbox{max} }}}$ (7.4%, ES = 1.14, *p* < 0.05) @ ~ 92% $\dot{V}{\text{O}}_{{2{ \hbox{max} }}}$ (5.9%, ES = 1.15, *p* < 0.05)	NS	2.6%, ES = 0.57, *p* < 0.01, NS G × T	–	–	–	Body mass, NS
Paavolainen et al. [73]	MVC knee extension (7.1%, *p* < 0.01), 5BJ (4.6%, *p* < 0.01)	@15 km h^−1^ (8.1%, ES = 3.22, *p* < 0.001) @13.2 km h^−1^, NS $\dot{V}{\text{O}}_{2}$@LT, NS	C: (4.9%, *p* < 0.05) $\dot{V}{\text{O}}_{{2{ \hbox{max} }}}$ demand (3.7%, *p* < 0.05, NS G × T)	–	–	5 km TT (3.1%, *p* < 0.05)	v20 m (3.4%, ES = 0.77, *p* < 0.01) vMART (ES = 1.98, *p* < 0.001)	Body mass, fat %, calf and thigh girth, NS
Pellegrino et al. [91]	CMJ (5.2%, *p* = 0.045, NS G × T)	@10.6 km h^−1^ (1.3%, *p* < 0.05 group) NS G × T @7.7, 9.2, 12.1, 13.5, 15.0, 16.4 km h^−1^, NS.	5.2%, ES = 0.49, *p* = 0.03, NS G × T	–	sLT, NS	3 km TT (2.6%, ES = 0.20, *p* = 0.04)	–	–
Piacentini et al. [86]	1RM leg press (HRT: 17%, ES = 0.69, *p* < 0.05), CMJ (C: 7%, ES = 0.63, *p* < 0.05), SJ (C: 13%, ES = 0.83, *p* < 0.01), Stiffness (RT: 13%, ES = 0.64, p < 0.05)	@10.75 km h^−1^/marathon pace (HRT: 6.2%, p < 0.05). @9.75,11.75 km h^−1^, NS	–	–	–	–	–	Body mass, fat mass, FFM, RMR, NS
Ramírez-Campillo et al. [87]	CMJ (8.9%, ES = 0.51, *p* < 0.01), DJ @20 cm (12.7%, ES = 0.43, *p* < 0.01), DJ @40 cm (16.7%, ES = 0.6, *p* < 0.05)	–	–	–	–	2.4 km TT (3.9%, ES = 0.4, *p* < 0.05)	20 m sprint (2.3%, ES = 0.3, *p* < 0.01)	Body mass, NS
Saunders et al. [77]	SJ RFD and peak force, NS. 5CMJ, NS	@18 km h^−1^ (4.1%, ES = 0.35, *p* < 0.05) @14,16 km h^−1^, NS	NS	–	BL @14,16,18 km h^−1^, NS	–	–	Body mass, NS
Schumann et al. [90, 92]	1RM leg press (I: NS, C: –4.7%, *p* = 0.011), MVC leg flexion (–9.7%, *p* = 0.031, ES = 0.96, NS G × T), MVC leg press NS, MVC knee ext. NS, CMJ NS	–	–	–	BL during 6 × 1 km (I: NS, C:, 21%, NS G × T) v4 mmol L^−1^ (I: 6%, C: 8%, NS G × T).	1 km TT after 5x 1 km, 60 s rec. (I: 9%, C: 13%, NS G × T)	–	Body mass, NS; CSA vastus lateralis (group diff. I: 7%, C: -6%, NS G × T); Total and leg lean mass (I: 2%, NS G × T)
Skovgaard et al. [88]	1RM squat (wk 4: 3.8%, wk 8: 12%, *p* < 0.001); 1RM leg press (wk 4: 8%, *p* < 0.05; wk 8: 18%, *p* < 0.001), 5RM deadlift (wk 4: 14%, wk8: 22%, *p* < 0.001)	@12 km h^−1^ (wk 8: 3.1%, ES = 1.53, *p* < 0.01)	NS	–	–	10 km TT (wk 4: 3.8%, ES = 1.50, *p* < 0.05) 1500 m TT (wk 8: 5.5%, ES = 0.67, *p* < 0.001)	–	Body mass, NS
Spurrs et al. [75]	MTS @75% MVC (left: 14.9%, right: 10.9%, *p* < 0.05), Calf MVC (left: 11.4%, right: 13.6%, *p* < 0.05). RFD NS	@12 km h^−1^ (6.7%, ES = 0.45), 14 km h^−1^ (6.4%, ES = 0.45), 16 km h^−1^ (4.1%, ES = 0.30), all *p* < 0.01	NS	–	–	3 km TT (2.7%, ES = 0.13,* p* < 0.05, NS G × T)	–	Body mass, NS
Støren et al. [79]	1RM (33.2%, *p* < 0.01) and RFD (26%, *p* < 0.01) half–squat	@70% $\dot{V}{\text{O}}_{{2{ \hbox{max} }}}$ (5%, ES = 1.03, *p* < 0.01)	NS	–	sLT, LT %$\dot{V}{\text{O}}_{{2{ \hbox{max} }}}$, NS	–	–	Body mass, NS
Turner et al. [76]	CMJ and SJ, NS	Ave. of 3 speeds: *M* = 9.6, 11.3, 12.9, *F* = 8.0, 9.6, 11.3 km h^−1^ (2–3%, *p* ≤ 0.05) @9.6 km h^−1^, NS	–	–	–	–	–	–
Vikmoen et al. [32, 38]	1RM half–squat (45%, ES = 2.4, *p* < 0.01), SJ (8.9%, ES = 0.83, *p* < 0.05), CMJ (5.9%, ES = 0.65, *p* < 0.05)	@10 km h^−1^, NS	NS	NS	v3.5 mmol L^−1^, NS	5 min TT (4.7%, ES = 0.95, *p* < 0.05). 40 min TT, NS		I: Leg mass (3.1%, ES = 1.69, p=*p* < 0.05), body mass, NS C: Leg mass (-2.2%), body mass decrease (-1.2%, *p* < 0.05)

*ARD* anaerobic running distance, *BJ* broad jump, *BL* blood lactate, *CMJ* counter-movement jump, *C* control group, *DJ* drop jump, *DJ*_*RSI*_ drop jump reactive strength index, *EC* energy cost, *EMG* electromyography, *ERT* explosive resistance training, *FFM* fat-free mass, *FU* fractional utilization, *GCT* ground contact time, *GRF* ground reaction force, *HR* heart rate, *HRT* heavy resistance training, *I* intervention group, k_leg_ leg stiffness, k_vert_ vertical stiffness, *(s)LT* (speed at) lactate threshold, *MAS* maximal aerobic speed, *MTS* musculotendinous stiffness, *MVC* maximum voluntary contraction, *PPO* peak power output, *PT* plyometric training, *QF* quadriceps femoris, *RCP* respiratory compensation point (*V*_E_/VCO_2_), *RFD* rate of force development, *RM* repetition maximum, *RMR* resting metabolic rate, *RT* resistance training, *RT*_*WBV*_ resistance training with whole body vibration, *SJ* squat jump, *TT* time trial, *TTE* time to exhaustion, *v* velocity, *vMART* velocity during maximal anaerobic running test, $\dot{V}{\text{O}}_{2}$ oxygen uptake, $\dot{V}{\text{O}}_{{2{ \hbox{max} }}} /\dot{V}{\text{O}}_{{2{\text{peak}}}}$ highest oxygen uptake associated with a maximal aerobic exercise test, *v* $\dot{V}{\text{O}}_{{2{ \hbox{max} }}}$ velocity associated with $\dot{V}{\text{O}}_{{2{ \hbox{max} }}}$, *wk* week

### Running Economy

An assessment of RE was included in all but four [31, 85, 87, 90, 92] of the studies in this review (Table 3). Running economy was quantified as the oxygen cost of running at a given speed in every case, except in three studies where a calculation of energy cost was used [82, 84, 91]. Statistically significant improvements (2–8%, ES: 0.14–3.22) in RE were observed for at least one speed in 14 papers. A single measure of RE was reported in four of these papers [31, 79, 80, 88], and a further four studies assessed RE across multiple different speeds and found improvements across all measures taken [72, 74, 75, 84]. Six papers reported a mixture of significant and non-significant results from the intensities they used to evaluate RE [36, 73, 76–78, 86]. Six studies failed to show any significant improvements in RE compared to a control group [32, 81–83, 89, 91].

### Maximal Oxygen Uptake

No statistically significant changes were reported in $\dot{V}{\text{O}}_{{2{ \hbox{max} }}}$ or $\dot{V}{\text{O}}_{{2{\text{peak}}}}$ for any group in the majority of studies that assessed this parameter [31, 32, 36, 72, 74, 75, 77–80, 85, 88, 89]. Three papers observed improvements for $\dot{V}{\text{O}}_{{2{ \hbox{max} }}}$ in the intervention group, but the change in score did not differ significantly from that of the control group [33, 81, 91]. One study detected a significant improvement (4.9%) in $\dot{V}{\text{O}}_{{2{ \hbox{max} }}}$ for the control group compared to the intervention group [73].

### Velocity Associated with $\dot{V}{\text{O}}_{{2{ \hbox{max} }}}$

Nine studies provided data on v$\dot{V}{\text{O}}_{{2{ \hbox{max} }}}$ or a similar metric [31–33, 36, 74, 78, 80, 85, 89]. Just two of these papers reported statistically significant improvements (3–4%, ES: 0.42–0.49) in the ST group compared to the control group [80, 89]. One study [74] reported a 2.6% improvement (ES: 0.57) and another [33] a 4.0% increase (ES: 0.9) after a 40-week intervention; however, these changes were not significantly different to the control group.

### Blood Lactate Parameters

Blood lactate value was measured at fixed velocities in six studies [77, 78, 81, 82, 84, 92] and velocity assessed for fixed concentrations of BL (2–4 mmol L^−1^) or lactate threshold (LT) in six studies [32, 33, 79, 81, 90, 91]. One study using young participants observed significantly greater improvements (11–12%) at two speeds compared to the control group [78]. Other studies found no significant changes following the intervention [32, 33, 77, 79, 82, 84, 91] or a change which was not superior to the control group [81, 90, 92].

### Time-Trial Performance

To assess the impact of ST directly upon distance running performance, studies utilized time trials over 1000 m (preceded by 5 × 1 km) [90, 92], 1500 m [88], 2.4 km [87], 3 km [75, 80, 91], 5 km [31, 73], 10 km [88, 89], 5 min [32], and 40 min [38]. There were similarities to competitive scenarios in most studies, including performances taking place under race conditions [31, 75, 87, 90–92], on an outdoor athletics track [31, 87–89], on an indoor athletics track [73, 75, 80, 90–92], and following a prolonged (90-min) submaximal run [38]. Performance improvements were statistically significant compared to a control group for eight of the 12 trials. The exceptions were a 40-min time trial [38], a 1000-m repetition [90, 92], and two studies that used a 3 km time trial [75, 80]. Statistically significant 3 km improvements were observed for all groups in one case [80]; however, the ES was larger for the two intervention groups (0.37 and 0.46) compared to the control group (0.20). Improvements over middle-distances (1500–3000 m) were generally moderate (3–5%, ES: 0.4–1.0). Moderate to large effects (ES: > 1.0) were observed for two studies [31, 88] that evaluated performance over longer distances (5–10 km); however, the relative improvements were quite similar (2–4%) over long distances compared to shorter distances [31, 73, 88, 89].

### Anaerobic Outcomes

Tests relating to anaerobic determinants of distance running performance were used in five investigations. Sprint speed over 20 m [73, 87] and 30 m [78] showed statistically significant improvements following ST (1.1–3.4%). Two studies provided evidence for enhancement of vMART [73, 78], and one further study showed no change in anaerobic running distance after 6 weeks of HRT [31]. A 30-s Wingate test was also used in one paper; however, no differences in performance were noted [89].

### Body Composition

Body mass did not change from baseline in 18 of the studies [32, 33, 36, 38, 72–75, 77, 79–81, 83, 84, 86–89]; however, one investigation reported a significant increase (2%, ES: 0.32) following ST [78]. This study also documented changes in the thickness of quadriceps femoris muscle in both the intervention (3.9%, ES: 0.35) and control group (1.9%, ES: 0.10) [78]. Similarly, an increase in total lean mass (3%) and leg lean mass (3%) was found following 12 weeks of ST despite little alteration in cross-sectional area of the vastus lateralis and body mass being noted [90, 92]. Another study observed a significant decrease (− 1.2%) in body mass in the control group, with no change in the intervention group [32]. A significant increase in leg mass (3.1%, ES: 1.69) was also noted in this study [32, 38]. Other indices of body composition that exhibited no significant changes were: fat mass [33, 36, 72, 73, 78, 86], fat-free mass [36, 72, 86], lean muscle mass [33, 78], skinfolds [83, 89], and limb girth measurements [72, 73, 83].

## Discussion

The aim of this systematic review was to identify and evaluate current literature which investigated the effects of ST exercise on the physiological determinants of middle- and long-distance running performance. The addition of new research published in this area, and the application of more liberal criteria provided results for 50% more participants (*n* = 469) compared to a recent review on RE [10]. Based upon the data presented herein, it appears that ST activities can positively affect performance directly and provide benefits to several physiological parameters that are important for distance running. However, inconsistencies exist within the literature, that can be attributed to differences in methodologies and characteristics of study participants, thus practitioners should be cautious when applying generalized recommendations to their athletes. Despite the moderate PEDro scores (4, 5, or 6), the quality of the works reviewed in this paper are generally considered acceptable when the unavoidable constraints imposed by a training intervention study (related to blinding) are taken into account.

### Running Economy

Running economy, defined as the oxygen or energy cost to run at a given sub-maximal velocity, is influenced by a variety of factors, including force-related and stretch–shortening cycle qualities, which can be improved with ST activities. In general, an ST intervention, lasting 6–20 weeks, added to the training program of a distance runner appears to enhance RE by 2–8%. This finding is in agreement with previous meta-analytical reviews in this area that show concurrent training has a beneficial effect (~ 4%) on RE [10, 26]. In real terms, an improvement in RE of this magnitude should theoretically allow a runner to operate at a lower relative intensity and thus improve training and/or race performance. No studies attempted to demonstrate this link directly, although inferences were made in studies, which noted improvements in RE and performance separately [73, 80, 88]. Other works provide evidence that small alterations in RE (~ 1.1%) directly translate to changes (~ 0.8%) in sub-maximal [94] and maximal running performance [95]. The typical error of measurement of RE has been reported to be 1–2% [96–99] and the smallest worthwhile change ~ 2% [94, 98, 100], which is thought to represent a “real” improvement and not simply a change due to variability of the measure. Taken together, it is therefore likely that the improvements seen in RE following a period of concurrent training would represent a meaningful change in performance.

Improvements were observed in moderately-trained [72, 76, 84, 86], well-trained [33, 36, 73, 75, 79, 80, 88] and highly-trained participants [74, 77], suggesting runners of any training status can benefit from ST. Different modes of ST were utilized in the studies, with RT or HRT [72, 78, 79, 84, 86], ERT [80], PT [75, 76, 80], and a combination of these activities [33, 36, 77], all augmenting RE to a similar extent. Single-joint isometric RT may also provide a benefit if performed at a high frequency (4 day week^−1^) [84]. Several studies adopted a periodized approach to the types of ST prioritized during each 3- to 6-week cycle [33, 36, 77, 88], which is likely to provide the best strategy to optimize gains long-term [101].

Six studies [32, 81–83, 89, 91] failed to show any improvement in RE and a further six [36, 73, 76–78, 86] observed both improvements and an absence of change at various velocities. This implies benefits are more likely to occur under specific conditions relating to the choice of exercises, participant characteristics, and velocity used to measure RE. In most studies that observed a benefit, exercises with free weights were utilized [33, 36, 72, 74, 86, 88]. Multi-joint exercises using free weights are likely to provide a superior neuromuscular stimulus compared to machine-based or single-joint exercises as they demand greater levels of co-ordination, multi-planar control, activation of synergistic muscle groups [102, 103] and usually require force to be produced from closed-kinetic chain positions. These types of exercise also have a greater biomechanical similarity to the running action so are therefore likely to provide a greater level of specificity and hence transfer of training effect [104]. An insufficient overload or a lack of movement pattern specificity may therefore be the reason for the absence of an effect in studies that used only resistance machines [32, 81] or a single-joint exercise [82]. These studies were also characterized by a lower frequency of sessions compared to studies that used similar RT exercises but did observe an improvement in RE [78, 84].

Moderately-trained runners were used in three of the six studies showing an absence of effect [81, 83, 91] and one used triathletes who performed a relatively low volume of running (34.8 km week^−1^) as part of their training [83]. However, a similar number of studies who used recreational athletes did show a positive effect [72, 76, 84, 86], suggesting that training level is unlikely to be the reason for the lack of response in these studies. This is also confirmed by recent observations that showed improvement in RE following a period of concurrent training was similar across individuals irrespective of training status and the number of sessions per week ST was performed [10].

The velocity used to assess RE may also explain the discrepancies in results across studies. It has been suggested that runners are most economical at the speeds they practice at most [98], and for investigations that utilized PT, stretch–shortening cycle improvements are likely to manifest at high running speeds where elastic mechanisms have greatest contribution [83, 105]. Therefore a velocity-specific measurement of RE may be the most valid strategy to establish whether an improvement has occurred. For example, Saunders and associates [77] observed an improvement (*p* = 0.02, ES: 0.35) at 18 km h^−1^ in elite runners, but an absence of change at slower speeds. Similarly, Millet and colleagues [74] noted large (ES: > 1.1) improvements at speeds faster than 75% v$\dot{V}{\text{O}}_{{2{ \hbox{max} }}}$ (~ 15 km h^−1^) in highly-trained triathletes, and Paavolainen et al. [73] detected changes at 15 km h^−1^ but not slower speeds in well-trained runners. Furthermore, Piacentini and co-workers [86] found improvement at race-pace in recreational marathon runners but not at a slower and a faster velocity. Improvements observed at faster compared to slower speeds may also reflect improvements in motor unit recruitment as a consequence of ST. As running speed increases there is a requirement for greater peak vertical forces due to shorter ground contact times, which elevates metabolic cost [25]. To produce higher forces, yet overcome a reduction in force per motor unit as a consequence of a faster shortening velocity, more motor unit recruitment is required [106]. Thus, an increase in absolute motor unit recruitment following a period of ST would result in a lower relative intensity reducing the necessity to recruit higher threshold motor units during running [25]. Several studies that failed to show any response used a single velocity to assess RE [32, 83, 89], perhaps indicating that the velocity selected was unsuitable to capture an improvement. Furthermore, only a small number of studies used relative speeds [33, 74, 79, 81, 82], with most choosing to assess participants at the same absolute intensity. A given speed for one runner may represent a high relative intensity, whereas for another runner it may be a relatively low intensity. Therefore selecting the same absolute speed in a group heterogeneous with respect to $\dot{V}{\text{O}}_{{2{ \hbox{max} }}}$, may not provide a true reflection of any changes which take place following an intervention. Moreover, this may also confound any potential improvements observed in fractional utilization of $\dot{V}{\text{O}}_{{2{ \hbox{max} }}}$.

Several common procedural issues exist in the studies reviewed, which may influence the interpretation of results and therefore conclusions drawn. The majority of studies quantified RE and $\dot{V}{\text{O}}_{{2{ \hbox{max} }}}$ as a ratio to body mass; however, oxygen uptake does not show a linear relationship with increasing body size [107]. It is also known that the relationship between body size and metabolic response varies across intensities, with a trend for an increasing size exponent as individuals move from low-intensity towards maximal exercise [108, 109]. Moreover, allometric scaling is likely to decrease interindividual variability [110], potentially improving the reliability of observations [99]. Ratio-scaling RE for all velocities to body mass is therefore theoretically and statistically inappropriate [111]. Just two studies [79, 80] used an appropriate allometric scaling exponent (0.75) to account for the non-linearity associated with oxygen uptake response to differences in body mass, both establishing a large ES in their results. The unsuitability of ratio-scaling as a normalization technique when processing physiological data is likely to have influenced the statistical outcomes of some studies and thus inaccurate conclusions may have been generated.

Running economy was expressed as oxygen cost in all but three studies [82, 84, 91], which quantified RE using the energy cost method. As the energy yield from the oxidation of carbohydrates and lipids differs, subtle alterations in substrate utilization during exercise can confound measurement of RE when expressed simply as an oxygen uptake value. Energy cost is therefore the more valid [112, 113] and reliable [99] metric for expressing economy, compared to traditional oxygen cost, as metabolic energy expenditure can be calculated using the respiratory exchange ratio, thus accounting for differences in substrate utilization. Despite attempts to control for confounding variables such as diet and lifestyle in most studies, equivalence in inter-trial substrate utilization cannot be guaranteed, which may have impacted upon the measurement of RE.

### Maximal Oxygen Uptake

Maximal oxygen uptake is widely regarded as one of the most important factors in distance running success [114], therefore the objective for any distance runner is to maximize their aerobic power [9]. An individual’s $\dot{V}{\text{O}}_{{2{ \hbox{max} }}}$ is limited by their ability to uptake, transport and utilize oxygen in the mitochondria of working muscles. Endurance training involving prolonged continuous bouts of exercise or high intensity interval training induces adaptations primarily within the cardiovascular and metabolic systems that results in improvements in $\dot{V}{\text{O}}_{{2{ \hbox{max} }}}$ [9, 115]. Conversely, ST is associated with a hypertrophy response that increases body mass and has been reported to decrease capillary density, oxidative enzymes and mitochondrial density [116–118], which would adversely impact aerobic performance. Theoretically there is therefore little basis for ST as a strategy to enhance aerobic power. However it is important to address whether in fact $\dot{V}{\text{O}}_{{2{ \hbox{max} }}}$ is negatively affected when distance running is performed concurrently with ST.

Thirteen works in this review found no change in $\dot{V}{\text{O}}_{{2{ \hbox{max} }}}$ following the intervention period, demonstrating that although ST does not appear to positively influence $\dot{V}{\text{O}}_{{2{ \hbox{max} }}}$, it also does not hinder aerobic power. Although ST in most studies was supplementary to running training, it appears that the additional physiological stimulus provided by ST was insufficient to elicit changes in cardiovascular-related parameters [119]. Three studies did observe significant increases in aerobic power that did not differ to the change observed in the control group [33, 81, 91], and one further study found an improvement in $\dot{V}{\text{O}}_{{2{ \hbox{max} }}}$ in the control group only [78]. It is perhaps surprising that more studies did not find an increase in $\dot{V}{\text{O}}_{{2{ \hbox{max} }}}$ (in any group) given that participants continued their normal running training through the study period. Improvements in $\dot{V}{\text{O}}_{{2{ \hbox{max} }}}$ of 5–10% have been shown following relatively short periods (< 6 weeks) of endurance training [9]; however, the magnitude of changes is dependent upon a variety of factors including the initial fitness level of individuals and the duration and nature of the training program [120]. Maximal oxygen uptake is known to have an innate upper limit for each individual, therefore in highly-trained and elite runners, long-term performance improvement is likely to result from enhancement of other physiological determinants, such as RE, fractional utilization and v$\dot{V}{\text{O}}_{{2{ \hbox{max} }}}$ [4, 121, 122]. A number of studies used moderately-trained participants [23, 72, 76, 81, 91], who would be the most likely to show an improvement in $\dot{V}{\text{O}}_{{2{ \hbox{max} }}}$ following a 6- to 14-week period of running, with two investigations demonstrating improvements for both groups [81, 91]. The absence of $\dot{V}{\text{O}}_{{2{ \hbox{max} }}}$ improvement in other papers suggests that the duration of the study and/or the training stimulus, was insufficient to generate an improvement [120]. Indeed, one study of 40 weeks’ duration in Collegiate level runners observed similar improvements (ES: 0.5–0.6) in $\dot{V}{\text{O}}_{{2{ \hbox{max} }}}$ in both groups [33], suggesting a longer time period may be required to detect changes in runners with a higher training status. High-intensity aerobic training (> 80% $\dot{V}{\text{O}}_{{2{ \hbox{max} }}}$) is a potent stimulus for driving changes in $\dot{V}{\text{O}}_{{2{ \hbox{max} }}}$[123]; however, some studies reported runners predominantly utilized low-intensity (< 70% $\dot{V}{\text{O}}_{{2{ \hbox{max} }}}$) continuous running [74, 78, 89], which may also explain the lack of changes observed.

### Velocity Associated with $\dot{V}{\text{O}}_{{2{ \hbox{max} }}}$

An individual’s v$\dot{V}{\text{O}}_{{2{ \hbox{max} }}}$ is influenced by their $\dot{V}{\text{O}}_{{2{ \hbox{max} }}}$, RE and anaerobic factors including neuromuscular capacity [4, 124]. The amalgamation of several physiological qualities into this single determinant appears to more accurately differentiate performance, particularly in well-trained runners [3, 98, 125, 126], therefore v$\dot{V}{\text{O}}_{{2{ \hbox{max} }}}$ has been labelled as an important endurance-specific measure of muscular power [127].

Improvements for v$\dot{V}{\text{O}}_{{2{ \hbox{max} }}}$ (3–4%, ES: 0.42–0.49) were found in two investigations [80, 89], with a further two studies observing improvements (2.6–4.0%, ES: 0.57–0.9) that could not be ascribed to the training differences between the groups [33, 74]. A number of studies also found little change in v$\dot{V}{\text{O}}_{{2{ \hbox{max} }}}$ following an intervention [31, 32, 36, 78, 85]. As v$\dot{V}{\text{O}}_{{2{ \hbox{max} }}}$ is the product of the interaction between aerobic and anaerobic variables, a small improvement in one area of physiology may not necessarily result in an increase in v$\dot{V}{\text{O}}_{{2{ \hbox{max} }}}$. Damasceno et al. [89] found an improvement in v$\dot{V}{\text{O}}_{{2{ \hbox{max} }}}$ (2.9%, *p* < 0.05, ES: 0.42) despite detecting no change in $\dot{V}{\text{O}}_{{2{ \hbox{max} }}}$, RE or Wingate performance, therefore attributed the change to the large improvements (23%, ES: 1.41) in the force-producing ability they observed in participants. Conversely, Berryman and associates [80] found changes in v$\dot{V}{\text{O}}_{{2{ \hbox{max} }}}$ (4.2%, ES: 0.43–0.49) alongside improvements in RE (4–7%, ES: 1.01), moderate increases in power output, and no change in $\dot{V}{\text{O}}_{{2{ \hbox{max} }}}$ scores. Beattie and co-workers [33] credited the change in v$\dot{V}{\text{O}}_{{2{ \hbox{max} }}}$ they observed (20-weeks: 3.5%, ES: 0.7) to the accumulation of improvements in RE, $\dot{V}{\text{O}}_{{2{ \hbox{max} }}}$ and anaerobic factors; however, these were not sufficiently large enough to provide a significant group × time interaction. Millet and colleagues [74] found notable improvements in RE (7.4%, ES: 1.14); however, changes in RE could not explain the changes observed in v$\dot{V}{\text{O}}_{{2{ \hbox{max} }}}$ (*r* = − 0.46, *p* = 0.09). It may also be the case that longer periods of ST are required before an improvement in v$\dot{V}{\text{O}}_{{2{ \hbox{max} }}}$ is detected, as studies showing an improvement (2.6–4.0%, ES: 0.57–0.9) from baseline lasted 14 weeks or more [33, 74], and studies showing little change tended to be 6–8 weeks in duration [31, 78, 85].

The conflicting results could also be explained by the inconsistency in methods used to define v$\dot{V}{\text{O}}_{{2{ \hbox{max} }}}$. A number of different protocols and predictive methods have been suggested to assess v$\dot{V}{\text{O}}_{{2{ \hbox{max} }}}$ [4], including determination from the $\dot{V}{\text{O}}_{2}$-velocity relationship [128] and the peak running speed attained during a maximal test using speed increments to achieve exhaustion [21, 127]. All studies that measured v$\dot{V}{\text{O}}_{{2{ \hbox{max} }}}$ in this review did so via an incremental run to exhaustion progressed using velocity. Velocity at $\dot{V}{\text{O}}_{{2{ \hbox{max} }}}$ was taken as the highest speed that could be maintained for a full 60-s stage [78, 80, 85], an average of the final 30-s [31, 36], the mean velocity in the final 120-s [32], or the minimum velocity that elicited $\dot{V}{\text{O}}_{{2{ \hbox{max} }}}$ [33, 74]. Although a direct approach to the measurement of v$\dot{V}{\text{O}}_{{2{ \hbox{max} }}}$ has been recommended [4], due to the velocity increments (0.5–1.0 km h^−1^) used in these investigations, this may not provide sufficient sensitivity to detect a change following a short- to medium-term intervention. Damasceno and associates [89] calculated v$\dot{V}{\text{O}}_{{2{ \hbox{max} }}}$ using a more precise method based upon the fractional time participants reached through the final stage of the test multiplied by the increment rate. This perhaps provided a greater level of accuracy which allowed the authors to identify the differences in changes which existed between the groups. Taken together, there is weak evidence that v$\dot{V}{\text{O}}_{{2{ \hbox{max} }}}$ can be improved following an ST intervention, despite constituent physiological qualities often exhibiting change. Differences in the protocols used to determine v$\dot{V}{\text{O}}_{{2{ \hbox{max} }}}$ makes comparison problematic; however, a more precise measurement of v$\dot{V}{\text{O}}_{{2{ \hbox{max} }}}$ that accounts for partial completion of a final stage is likely to provide the sensitivity to identify subtle changes that may occur.

The critical velocity model, which represents exercise tolerance in the severe intensity domain, potentially offers an alternative to measurement of v$\dot{V}{\text{O}}_{{2{ \hbox{max} }}}$ that is currently uninvestigated in runners [35, 129]. Two main parameters can be assessed using the critical velocity model; critical velocity itself, which is defined as the lower boundary of the severe intensity domain which when maintained to exhaustion leads to attainment of $\dot{V}{\text{O}}_{{2{ \hbox{max} }}}$, and the curvature constant of the velocity–time hyperbola above critical velocity, which is represented by the total distance that can be covered prior to exhaustion at a constant velocity [130]. Middle-distance running performance (800 m) is strongly related to critical velocity models (*r* = 0.83–0.94) in trained runners [131], and may be more important than RE in well-trained runners [35]. Evidence from studies using untrained participants has demonstrated that the total amount of work that can be performed above critical power during high-intensity cycling exercise is improved (35–60%) following 6–8 weeks of RT [132, 133]. Future investigations should therefore address the dearth in literature around how ST might positively influence parameters related to the critical velocity model [35].

### Blood Lactate Markers

A runner’s velocity at a reference point on the lactate-velocity curve (e.g., LT) or BL for a given running speed are important predictors of distance running performance [134–136]. A runners LT also corresponds to the fractional utilization of $\dot{V}{\text{O}}_{{2{ \hbox{max} }}}$ that can be sustained for a given distance [114], therefore an increase in LT also allows a greater proportion of aerobic capacity to be accessed.

In contrast to RE, ST appears to have little impact upon BL markers. This is quite surprising as an improvement in RE should theoretically result in an enhancement in speed for a fixed BL concentration. This suggests that adaptations to RE can occur independently to changes in metabolic markers of performance. An absence of change in BL also implies that ST does not alter anaerobic energy contribution during running, thus assuming aerobic energy cost of running is reduced following ST, it can be inferred that total energy cost (aerobic plus anaerobic energy) is also likely to be reduced. Previous studies have shown as little as 6 weeks of endurance training can improve BL levels or the velocity corresponding to an arbitrary BL value in runners [137–139]. The intensity of training is important to elicit improvement in BL parameters [140], therefore it appears that the running training prescription may have been insufficient to stimulate improvements, or the training status of participants meant a longer period was required to realize a meaningful change. In addition, the inter-session reliability of BL measurement between 2–4 mmol L^−1^ is ~ 0.2 mmol  L^−1^ [99], therefore over a short study duration this metric may not provide sufficient sensitivity to detect change.

Training at an intensity above the LT is likely to result in a reduction in the rate of BL production (and therefore accumulation), or an improved lactate clearance ability from the blood [9]. Short duration high-intensity bouts of activity generate high levels of BL so drive metabolic adaptations which can result in an improvement in performance [141–143]. Studies that have utilized high-repetition, low-load RT in endurance athletes therefore have the potential to produce high BL concentrations so may provide an additional stimulus to improve performance via BL parameters. This theory is supported by works that have demonstrated improvements in BL-related variables in endurance athletes following an intervention that uses a strength-endurance style of conditioning with limited rest between sets [54, 62, 144]. The ST prescription in the studies reviewed was predominantly low-repetition, high-intensity RT or PT, which is unlikely to have provided a metabolic environment sufficient to directly enhance adaptations related to BL markers.

### Time-Trial Performance

Physiological parameters such as $\dot{V}{\text{O}}_{{2{ \hbox{max} }}}$, v$\dot{V}{\text{O}}_{{2{ \hbox{max} }}}$, RE and LT are clearly important determinants that can be quantified in a laboratory; however, for a runner, TT performance possesses a far higher degree of external validity. Similar improvements in TT performance were observed for middle-distance events (3–5%, ES: 0.4–1.0) and long-distance events up to 10 km (2–4%, ES: 1.06–1.5). In the majority of these studies, time trials took place in a similar environment and under comparable conditions to a race, therefore these findings have genuine applicability to “real-life” scenarios. These improvements are likely to be a consequence of significant enhancements in one or more determinants of performance. Interestingly, Damasceno and co-authors [89] found an improvement in 10 km TT performance due to the attainment of higher speeds in the final 3 km, despite observing no change in RE during a separate assessment. This suggests that greater levels of muscular strength may result in lower levels of relative force production per stride, thereby delaying recruitment of higher threshold muscle fibers and thus providing a fatigue resistant effect [145]. This subsequently manifests in a superior performance during the latter stages of long-distance events [89].

Four studies observed no difference in performance change compared to a control group [38, 75, 80, 90, 92]. Vikmoen and colleagues [38] attributed a lack of effect in their 40 min TT to the slow running velocity caused by the 5.3% treadmill inclination used in the test. This was also the only study to use a treadmill set to a pre-determined velocity which participants could control once the test had commenced. The absence of natural self-pacing may therefore have prevented participants achieving their true potential on the test. Spurrs et al. [75] and Berryman et al. [80] both found improvements in 3 km performance compared to a pre-training measure of a comparable magnitude to other studies (2.7–4.8%, ES: 0.13–0.46); however, changes were not significantly different to a control group, suggesting ST provided no additional benefit or there was a practice effect associated with the test.

It could be possible that enhancement of physiological qualities in some studies could be attributed to RT being positioned immediately after low-intensity, non-depleting running sessions [146]. This arrangement of activities in concurrent training programs has been shown to provide a superior stimulus for endurance adaptation compared to performing separate sessions, and without compromising the signaling response regulating strength gains [147, 148]. This, however, appears not to be the case, as most studies reported ST activities took place on different days to running sessions [85, 88, 89] or were at least performed as separate sessions within the same day [33, 36, 38, 72, 75, 78]. Only three studies performed ST and running immediately after one another, with one positioning PT before running [87] and one lacking clarity on sequencing [76]. Schumann and colleagues [90, 92] observed no additional benefit to both strength and endurance outcomes compared to a running only group, when ST was performed immediately following an incremental running session (65–85% maximal heart rate), citing residual fatigue which compromised quality of ST sessions as the reason.

### Anaerobic Running Performance

The contribution of anaerobic factors to distance running performance is well established [127, 149]. In particular, anaerobic capacity and neuromuscular capabilities are thought to play a large role in discriminating performance in runners who are closely matched from an aerobic perspective [124, 150]. An individual’s v$\dot{V}{\text{O}}_{{2{ \hbox{max} }}}$ perhaps provides the most functional representation of neuromuscular power in distance runners; however, measures of maximal running velocity and anaerobic capacity are also potentially important [127].

Tests for pure maximal sprinting velocity (20–30 m) were used in three studies [73, 78, 87] and showed improvements (1.1–3.4%) following ST in every case. This confirms results from previous studies that have shown sprinting performance can be positively affected by an ST intervention in shorter-distance specialists [151–153]. This finding has important implications for distance runners, as competitive events often involve mid-race surges and outcomes are frequently determined in sprint-finishes, particularly at an elite level [154–157]. Middle-distance runners also benefit from an ability to produce fast running speeds at the start of races [158], therefore improving maximum speed allows for a greater “anaerobic speed reserve” [159], resulting in a lower relative work-rate, and thus decreasing anaerobic energy contribution [41]. Interestingly, endurance training in cyclists has been shown to improve critical power [160] but reduce work capacity for short duration exercise [161, 162]. It is unknown whether long-term aerobic training has a similar effect on anaerobic running qualities; however, ST offers a strategy to avoid this potential negative consequence.

The velocity attained during a maximal anaerobic running test provides an indirect measure of anaerobic and neuromuscular performance, and has a strong relationship (*r* = 0.85) to v$\dot{V}{\text{O}}_{{2{ \hbox{max} }}}$ [19]. The vMART is particularly relevant to middle-distance runners because it requires athletes to produce fast running speeds under high-levels of fatigue caused by the acidosis and metabolites derived from glycolysis [163]. Both studies that included this test observed significant improvements in vMART (1.1–3.4%), which can be attributed to changes observed in neuromuscular power as a result of the ST intervention [73, 78]. One study showed no alteration in the predicted distance achieved on an anaerobic running test following 6 weeks of HRT; however, the validity and reliability of the test was questioned by the authors [31]. Performance on a 30 s Wingate test was also unchanged following 8 weeks of running training combined with HRT in recreational participants [89]. This finding perhaps underlines the importance of selecting tests which are specific to the training which has been performed in the investigation.

### Strength Outcomes

Changes in strength outcomes were evident in most studies despite all but one [78] observing no change in body mass. Since strength changes can be ascribed to both neurological and morphological adaptations [164], it is therefore likely that improvements are primarily underpinned by alterations in intra- and inter-muscular co-ordination. It is also known that initial gains in strength in non-strength trained individuals are the consequence of neural adaptations rather than structural changes [118]. An improvement in force producing capability is perhaps expected in individuals who have little or no strength-training experience [165]; however, concurrent regimens of training have consistently been shown to attenuate strength-related adaptation [30].

The seminal paper published by Hickson et al. [48] was the first to identify the potential for endurance exercise to mitigate strength gains, when both training modalities were performed concurrently within the same program. Follow-up investigations have since shown mixed results [166–171], but evidence from this review clearly demonstrates that, for the distance runner at least, strength-related improvements are certainly possible following a concurrent period of training. Nevertheless, the study designs adopted by the works under review did not include a strength-only training group, thus it is not possible to determine whether strength adaptation was in fact negated under a concurrent regimen. One study using well-trained endurance cyclists with no ST experience, observed a blunted strength response in a group who added ST to their endurance training compared to a group who only performed ST [170]. Based upon this finding and other similar observations [167, 172, 173] it seems likely that although distance runners can significantly improve their strength using a concurrent approach to training, strength outcomes are unlikely to be maximized. Moreover, the degree of interference with strength-adaptation also appears to be exacerbated when volumes of endurance training are increased and the duration of concurrent training programs is longer [30, 146].

### Body Composition

Resistance training performed 2–3 times per week is associated with increases in muscle cross-sectional area as a principal adaptation [174]. Although gains in gross body mass may appear to be an unfavorable outcome for distance runners, the addition of muscle mass to proximal regions of the lower limb (i.e., gluteal muscles) should theoretically provide an advantage, via increases in hip extension forces, minimizing moment of inertia of the swinging limb, and reducing absolute energy usage [25]. It is somewhat surprising that virtually all studies demonstrated an absence of change in body mass, fat-free mass, lean muscle mass, and limb girths. Other than one investigation [33], the duration of the studies that observed no effect on measures of body composition was < 14 weeks, suggesting this may not have been sufficiently long to demonstrate a clear hypertrophic response. There is also a possibility that small increases in muscle mass within specific muscle groups (e.g., gluteals) were present, and contributed to the improvements observed in RE, but these may not have been detectable using a gross measure of mass. Evidence for this may have occurred in the Schumann et al. study [90, 92], who observed increases in total lean mass (3%) despite noting no significant change in body mass or cross-sectional area of the vastus lateralis compared to baseline measures.

The interference effect observed during concomitant integration of endurance and ST as part of the same program may also provide an explanation for the lack of change in measures of mass. Following a bout of exercise, a number of primary and secondary signaling messengers are up regulated for 3–12 h [175], which initiate a series of molecular events that serve to activate or suppress specific genes. The signaling messengers which are activated, relate to the specific stress which is imposed on the physiological systems involved in an exercise bout. Strength training causes mechanical perturbation to the muscle cell, which elicits a multitude of signaling pathways that lead to a hypertrophic response [176]. In particular, the secretion of insulin-like growth factor-1 as a result of intense muscular contraction is likely to cause a cascade of signaling events which increase activity of phosphoinositide-3-dependent kinase (Pl-3 k) and the mammalian target of Rapamycin (mTOR) [177–179]. There is strong evidence that mTOR is responsible for mediating skeletal muscle hypertrophy via activation of ribosome proteins which up regulate protein synthesis [180]. Prolonged exercise bouts, such as those associated with endurance training, activate metabolic signals related to energy depletion, uptake and release of calcium ions from the sarcoplasmic reticulum and oxidative stress in cells [181]. Adenosine monophosphate activated kinase (AMPK) is a potent secondary messenger which functions to monitor energy homeostasis [182] and when activated, modulates the release of peroxisome proliferator co-activator-1α, which along with calcium-calmodulin-dependent kinases increase mitochondrial function to enhance aerobic function [181, 183, 184]. Crucially though, AMPK also acts to inhibit the Pl-3 k/mTOR stage of the pathway via activation of the tuberous sclerosis complex thereby suppressing the ST induced up regulation of protein synthesis [185, 186]. This conflict arising at a molecular signaling level therefore appears to impair the muscle fiber hypertrophy response to ST and attenuate increases in body mass [186].

### Muscle–Tendon Interaction Mechanisms

The potential mechanisms for the positive changes observed in physiological parameters underpinning running performance were directly investigated in three studies [82, 84, 91], and were inferred from gait measures [36, 73–75, 77] and strength outcomes in others. It is well documented that muscle–tendon unit stiffness correlates well with RE [187–189]. Tendons are also highly adaptable to mechanical loading and have been shown to increase in stiffness in response to HRT and PT [84, 190, 191]. Despite observing no statistical effect for HRT on RE, Fletcher and colleagues [82] also found a relationship between the change in RE and the changes observed in Achilles tendon stiffness. Despite these associations, it is likely that improvements in RE are a consequence of the interaction between adaptations to tendon properties and improvements in motor unit activation which influence behavior of force–length-velocity properties of muscles [25]. It tends to be assumed that improved tendon stiffness allows the body to store and return elastic energy more effectively, which results in a reduction in muscle energy cost due to a greater contribution from the elastic recoil properties of tendons [192]. Indeed, authors of studies in the present review have argued that the improvements observed in RE following a period of ST are due to an enhanced utilization of elastic energy during running [36, 73–75]. An alternative proposal, based upon more recent evidence, suggests the Achilles tendon provides a very small contribution to the total energy cost of running therefore improvements in stiffness provide a negligible reduction in energy cost [193, 194]. Instead, a tendon with an optimal stiffness contributes to reducing RE by minimizing the magnitude and velocity of muscle shortening, thus allowing muscle fascicles to optimize their length and remain closer to an isometric state [25]. A reduction in the amount and velocity of fiber shortening therefore reduces the level of muscle activation required and hence the energy cost of running [193].

The improvements observed in maximal and explosive strength, which can be attributed to increases in motor unit recruitment and firing frequency, enable the lower limb to resist eccentric forces during the early part of ground contact [165] and thus contribute to the attainment of a near isometric state during stance. As the force required to sustain speed during distance running performance is submaximal, the level of motor unit activation needed can be minimized when fascicles contract isometrically [25]. This enables the Achilles tendon in particular to accommodate a greater proportion of the muscle–tendon unit length change during running thereby reducing metabolic cost [194]. Variables which provide an indirect measure of the neuromuscular systems ability to produce force rapidly and utilize tendon stiffness were found to improve in other studies that showed improvements in running performance and/or key determinants [73, 74, 78–80, 87]. However, some studies found improvements in running-related parameters despite observing no alterations in jump performance [33, 76–78, 91], rate of force development [36, 75, 77], or stiffness [33, 74, 89] illustrating that measures were insufficiently sensitive to detect change, or a combination of mechanisms is likely to be contributing towards the enhancements observed.

Heavy RT causes a shift in muscle fiber phenotype, from the less efficient myosin heavy chain (MHC) IIx to more oxidative MHC IIa, [195, 196]. A higher proportion of MHC IIa has been shown to relate to better running economy [91, 197, 198]; however, whether changes to MHC properties as a result of ST contribute to an improvement in RE and performance remains to be determined. One previous study provided evidence that 4 weeks of sprint running (30-s bouts) improve RE and also the percentage of MHC IIx [199]; however, the absence of endurance training may partly explain the shift in phenotype. Over a longer period (6 weeks), Pellegrino and co-workers [91] found no measurable changes in MHC isoforms following a PT intervention despite a significant improvement in 3 km TT performance, suggesting that a contribution from this mechanism is unlikely for distance running.

It could also be speculated that improvements in RE due to improved strength might have resulted in subtle changes to running kinematics, thus enabling participants to perform less work for a given submaximal speed [72]. There is currently little direct support for this conjecture; however, previous work has shown that running technique is an important component of RE [200, 201], and improving hip strength can reduce undesirable frontal and transverse plane motion in the lower limb during running [202]. One study in this review did observe a reduction in EMG amplitude in the superficial musculature of the lower limb following ST; however, this wasn’t accompanied by an improvement in RE [83]. This suggests that favorable adaptations in neuromuscular control do not necessarily translate to reducing the metabolic cost of running. Additionally, two studies showed significant increases (3.0–4.4%) in ground contact time during submaximal running after an ST intervention [36, 81]; however, only Giovanelli and colleagues [36] found a corresponding improvement in RE. Several papers have demonstrated an inverse relationship between RE and ground contact times [201, 203, 204], since a lower peak vertical force is required to generate the same amount of impulse during longer compared to short ground contacts [25]. Although there is currently minimal evidence to suggest an ST intervention increases ground contact time during sub-maximal running, this mechanism may in part explain the improvements in RE.

### Strength-Training Prescription

#### Modality and Exercise Selection

The works included in this review used a variety of ST modalities; however, the most effective type of training is currently difficult to discern. Adaptations are specific to the demands placed upon the body, therefore it would be expected that HRT, ERT and PT produce somewhat different outcomes [205]. This can be observed in the study by Berryman and co-workers [80], who observed larger improvements in explosive concentric power in a group following an ERT program compared to a group who used PT. The opposite result occurred for the counter-movement jump, which places a greater reliance on a plyometric action; the PT group displayed greater improvements than the ERT group [80]. Heavy RT, which is characterized by slow velocities of movement, is likely to improve agonist muscle activation via enhanced recruitment of the motor neuron pool, whereas ERT, which involves lighter loads being moved rapidly, tends to enhance firing frequency and hence improve rate of force development [164, 165]. Plyometric training develops properties related to the stretch–shortening cycle function [206], and uses movements patterns which closely mimic the running action (e.g., hopping and skipping). It is therefore likely that although a variety of ST methods are capable of improving physiological parameters relating to distance running performance, the mechanisms underpinning the response may differ.

In less strength-trained individuals, such as those used in the studies reviewed, any novel ST stimulus is likely to provide a sufficient overload to the neuromuscular system to induce an adaptation in the short term [207]. This is perhaps why ST is effective even in highly-trained distance runners [74, 77, 87]. Studies that have attempted to compare ST techniques in distance runners have generally shown HRT to be superior to ERT or a mixed methods approach at improving aerobic parameters [57, 63] and maximal anaerobic running speed [62]. Plyometric training has also shown superiority to ERT for improvement of RE in moderately trained runners [80]. Other investigations have found no differences in the physiological changes between groups using HRT, ERT or a mixture of modalities [62, 65]. A number of studies have also shown HRT and/or ERT to be more beneficial to a muscular endurance style of ST [59, 64, 65, 67, 86]. The addition of whole body vibration to RT also provides no extra benefit [85]. Although ERT and PT may have more appeal compared to HRT due to their higher-level of biomechanical similarity to running, an initial period of HRT is likely to provide an advantage long-term in terms of reducing injury risk [208] and eliciting a more pronounced training effect [209]. Taken together, it seems that long-term, a mixed modality approach to ST is most effective, as this provides the variety and continual overload required to ensure the neuromuscular system is constantly challenged. One study that used a longer intervention period lends support to this notion, as significant improvements were observed in strength and physiological measures after 20 and 40 weeks with a periodized methodology that used several types of ST [33]. Further research is required to ascertain the long-term benefits of various ST modalities and the relative merits of different approaches to sequencing and progressing these modalities.

As discussed in Sect. 4.1, the exercises selected in an ST program can potentially influence the magnitude of neuromuscular adaptation and thus the impact on physiological determinants of performance. Exercises using free weights, which require force to be generated from the leg extensor muscles in a close-kinetic chain position, are the most likely to positively transfer to running performance [210]. Examples of RT exercises commonly used include: barbell squat, deadlifts, step-ups and lunging movement patterns [31, 33, 36, 72, 79, 85, 88]. Isometric HRT may also have value for the plantarflexors [84]. Explosive RT, by its very nature, should avoid a deceleration phase, therefore exercises such as squat jumps and Olympic weightlifting derivatives should be utilized [33, 80]. To maximize transfer to distance running performance, particularly at faster speeds, PT exercises should exhibit short ground contact times (< 0.2 s) [36, 72], which approximates the contact times observed in competitive middle- [211] and long-distance running [212], and encourages a rapid excitation–contraction coupling sequence and improved musculotendinous stiffness [36, 73–75]. Exercises which possess a low to moderate eccentric demand such as depth jumps (from a 20–30 cm box), skipping, hopping, speed bounding appear most suitable [33, 73, 75, 77, 80, 83].

#### Intra-Session Variables

For non-strength trained individuals, exercise prescription and gradual progression is important to avoid injury and overtraining [213]. Most studies initially used 1–2 sets and progressed to 3–6 sets over the course of the intervention period for HRT, ERT and PT, which appears appropriate to circumvent these risks. Several studies utilized a low (3–5) repetition range in every HRT session [31, 79, 81, 86] at loads which approached maximum (≥ 80% 1RM or repetition failure), but did not observe superior benefits compared to investigations that prescribed RT at moderate loads (60–80% 1RM) and higher repetition ranges (5–15 repetitions). Sets were performed to RM in a number of studies [32, 38, 72, 79, 81, 88, 89], which was likely employed as a means of standardizing the intensity of each set in the absence of 1RM data for participants. Performing sets which leads to repetition failure induces a high level of metabolic and neuromuscular fatigue, which may delay recovery [214]. Although training to repetition failure may be more important than the load lifted for inducing a hypertrophy response [215], this is both unfavorable and unnecessary to optimize gains in strength compared to a non-repetition failure strategy [216]. Not working to repetition failure also appears to become a more important feature of RT as ST status increases [216]. Participants were often instructed to move the weights as rapidly as possible when performing the concentric phase of RT exercises, which increases the likelihood of maximizing neuromuscular adaptations [217]. Plyometric training is characterized by high eccentric forces compared to running and RT, therefore repetitions per set were typically low (4–10 repetitions). Total foot contacts progressed from 30 to 60 repetitions in the first week of an intervention up to 110–228 repetitions after 6–9 weeks [73, 75, 76, 91]. Plyometric exercises were all performed without additional external resistance in all but one study [73] and in many cases a short ground contact time [76, 77, 83] and maximal height [80, 83] were cued to amplify the intensity. An inter-set recovery period of 2–3 min was typical for HRT, ERT and PT, which is in line with recommendations for these training techniques [213]. Where SpT was incorporated into ST programs, repetition distances were short (20–150 m) and performed at or close to maximal running speed [73, 74, 88].

#### Inter-Session Variables

The majority of studies that demonstrated improvements in running physiology scheduled ST 2–3 times per week, which is in line with the guidelines for non-strength trained individuals [213]. One study used just one session per week (ERT or PT) and achieved moderate improvements in strength outcomes and RE after 8 weeks of training [80]. Beattie and associates [33] observed small improvements (ES: 0.3) in RE using a single ST session (mixed activities) each week for 20 weeks; however, the participants had already experienced moderate improvement (ES: 1.0) in this parameter using a twice weekly program in the 20 weeks prior. For well-trained runners who complete 8–13 running sessions per week [73, 77], it would be useful to establish the minimal ST dosage required to elicit a beneficial effect to reduce the risk of overtraining. Equally, for the recreational runner, ST may take up valuable leisure time that could be spent running, therefore identifying the optimal volume and frequency of ST to achieve an improvement in performance would be desirable. A previous meta-analysis indicated that two or three sessions per week provides a large effect on strength, but for the non-strength trained individual, three sessions is superior to two sessions per week [218]. More recently, a weak relationship was established between improvement in RE and weekly frequency of ST sessions in 311 endurance runners [10]. This suggests that higher weekly volumes of ST would not necessarily provide greater RE improvements, therefore two sessions per week is likely to be sufficient [10].

Given the volume of endurance training participants were exposed to and the duration of each study, it seems likely that an attenuation of strength-related adaptation would have occurred. To minimize this interference phenomenon, it is therefore recommended that a recovery period of > 3 h is provided following high-intensity running training before ST takes place [146]. In many studies running training and ST took place on different days [33, 36, 85, 88, 89], and several papers noted a gap of > 3 h between running and ST on the same day [32, 38, 72, 78, 79]. This feature of concurrent training prescription therefore appears important in ensuring sufficient strength-adaptations are realized but without compromising running training. Although there is very little evidence that the dosage of ST prescribed impaired any endurance-related adaptations, recent work has highlighted that acute bouts of RT may cause fatigue sufficient to impair subsequent running performance, which long term may result in sub-optimal adaptation [219]. It is therefore recommended that this potential fatigue is accounted for by allowing at least 24 h recovery between an ST session and an intensive running session [33, 85, 88, 89].

The results provide compelling evidence that a relatively short period (6 weeks) of ST can enhance physiological qualities related to distance running performance. Improvements in RE [57] and 10 km TT performance [88] have also been shown in as little as 4 weeks. A relationship between intervention duration and improvement in RE has previously been reported [10], suggesting that longer periods of ST provide a larger benefit. The same may be true for v$\dot{V}{\text{O}}_{{2{ \hbox{max} }}}$; however, more research using longer periods of ST is required to establish if this is indeed the case. The benefits to performance also seem to be dependent on study duration as most short interventions (6 weeks) tended to produce small TT improvements (2.4–2.7%, ES: 0.13–0.4) [75, 87, 91], whereas longer programs (8–11 weeks) resulted in moderate or large performance effects (3.1–5.5%, ES: 0.67–1.50) [32, 73, 88]. It would seem reasonable to assume that highly-trained distance runners would require a higher volume of ST to achieve the same benefit as less experienced runners; however, this does not appear to be the case. Relatively short (6–9 weeks) periods of ST improved RE and TT performance to a similar extent in highly-trained individuals [77, 87] and recreational runners [76, 86, 91]. It is therefore recommended that future investigations use periods of 10 weeks or longer to provide further insight into how ST modalities may impact physiological parameters long-term in different types of distance runner.

The time of year or phase of training when the research was conducted was not reported in the majority of studies. Several papers indicated that the intervention formed part of an off-season preparation period [73, 74, 78, 82, 86], but others scheduled the intervention within the competition period [32, 38, 87]. Based upon the literature reviewed, it is currently not possible to provide specific recommendations for ST in different phases of a runners training macrocycle, as most studies found at least some physiological or performance benefits to concurrent training. Importantly though, evidence suggests that choosing to exclude ST following a successful intervention period results in a detraining effect which causes improvements to return to baseline levels within 6 weeks [31]. The 40-week intervention conducted by Beattie and colleagues [33] provides evidence that reducing ST volume from two sessions per week (both with a lower limb HRT emphasis) during the preparatory phase to one weekly session (ERT and PT emphasis) during the in-season racing period is sufficient to at least maintain previous strength and physiological gains. This finding corroborates with a maintenance effect observed in cyclists [220, 221] and soccer players [222] showing one ST session per week is sufficient to preserve the strength qualities developed during a preceding phase of training. Therefore, runners can decrease ST volume from 2–3 sessions per week (each with a lower limb focus) in preparatory phases of training to a single session each week during the competitive season without fearing a loss of adaptation as a consequence of the reduction in training density.

It is currently uncertain what volume and intensity of running and ST are most likely to avoid the interference effect associated with concurrent training practices. One option to minimize attenuation of strength development is to organize activities into periods that concentrate on developing either strength or endurance adaptation [223]. This polarized approach to planning seems unnecessary and counterintuitive for distance runners who generally possess little ST experience, therefore require a minimal stimulus to create an adaptation. Indeed, studies that replaced running training with ST [73, 78, 88] found no greater benefit than those which included ST in a supplementary manner.

#### Training Supervision

In most studies, the ST routine was supervised and tightly monitored; however, similar controls were often absent for the running training participants performed. It seems reasonable to assume that any errors in participants training logbooks would be similar across intervention and control groups; however, validity of findings would be improved if the running component of training had been more tightly defined. Where supervision of the ST exercises was not included [76] or only included for the first 2 weeks [36], strength measures did not improve following the intervention period. This indicates that a suitably qualified coach is an important feature of an ST programme for a distance runner who lacks ST experience.

### Limitations

In addition to the limitations already highlighted in this review, there are other weaknesses that should be acknowledged. For many of the studies reviewed, calculation of an ES was possible for the variables measured, which provides insight into the meaningfulness and substantiveness of results. However, despite the qualitative nature of this review, interpretation of findings was predominantly based upon reported probability values, which can be misleading due to low sample sizes and the heterogeneity in the pool of participants studied. A relatively large number of studies have been included in this review; however, several parameters (e.g., v$\dot{V}{\text{O}}_{{2{ \hbox{max} }}}$ and BL) were measured in only a small number of studies, which increases the possibility that false conclusions may be drawn.

There was also a lack of detail concerning several important confounding variables in studies, such as the nature of running training prescription and participant’s previous experience in ST. All but seven studies [31, 73, 74, 76, 84, 86, 90, 92] identified that participants had not been engaged in a program of ST for at least 3 months prior to the study commencing. Although it is perhaps unlikely that participants in these seven studies were strength-trained, this cannot be discounted and may therefore have influenced findings in these investigations.

## Conclusion and Future Research

This review is the most comprehensive to date surrounding the potential impact of ST on the physiological determinants of distance running. The research reviewed suggests that supplementing the training of a distance runner with ST is likely to provide improvements to RE, TT performance and anaerobic parameters such as maximal sprint speed. Improvements in RE in the absence of changes in $\dot{V}{\text{O}}_{{2{ \hbox{max} }}}$, BL and body composition parameters suggests that the underlying mechanisms predominantly relate to alterations in intra-muscular co-ordination and increases in tendon stiffness which contribute to optimizing force–length-velocity properties of muscle. Nevertheless, it is clear that the inclusion of ST does not adversely affect $\dot{V}{\text{O}}_{{2{ \hbox{max} }}}$ or BL markers. The addition of two to three supervised ST sessions per week is likely to provide a sufficient stimulus to augment parameters within a 6- to 14-week period, and benefits are likely to be larger for interventions of a longer duration. A variety of ST modalities can be used to achieve similar outcomes assuming runners are of a non-strength trained status; however, to maximize long-term adaptations, it is suggested that a periodized approach is adopted with HRT prioritized initially. Although changes in fat-free mass were not observed in the majority of studies, a targeted RT program, which aims to increase muscle mass specifically around the proximal region of the lower limb may enhance biomechanical and physiological factors which positively influence RE.

A number of methodological issues are likely to have contributed towards the discrepancies in results and should be acknowledged in future research conducted in this area. In particular, the measurement of RE should be quantified as energy cost (rather than oxygen cost) and a variety of speeds assessed which are relative to the maximum steady state of each participant. Furthermore, when quantifying RE and $\dot{V}{\text{O}}_{{2{ \hbox{max} }}}$, differences in body size should be accounted for by using scaling exponents which are appropriate for the cohort under investigation. Although a direct measure of v$\dot{V}{\text{O}}_{{2{ \hbox{max} }}}$ has obvious validity, the discrete increments utilized during a maximal test may not provide the sensitivity required to detect changes which exist in this parameter following a relatively short intervention. Alternative strategies to quantifying v$\dot{V}{\text{O}}_{{2{ \hbox{max} }}}$ may provide a solution. It is therefore recommended that future studies focus their time and efforts on investigating the effects of ST on physiological variables other than $\dot{V}{\text{O}}_{{2{ \hbox{max} }}}$ and BL responses, such as RE, v$\dot{V}{\text{O}}_{{2{ \hbox{max} }}}$ and parameters associated with the critical power model. The nature of the running training undertaken by participants and strength training history potentially confounds the outcomes of studies in this area, therefore attempts should also be made to control these variables as much as possible.

Although the interference phenomenon is likely to have blunted the strength adaptations observed, the extent to which this occurs is currently uncertain due to the absence of a strength-only training group in the studies reviewed. For longer term interventions, where improvements inevitably plateau, minimizing attenuation to strength outcomes (and equally augmenting aerobic adaptation) potentially becomes more important. Therefore the organization of ST around running training provides a further avenue for investigation. Similarly, it would be useful for practitioners to understand the optimal sequencing of ST modalities within a long-term program in order to optimize training outcomes and facilitate a peaking response. Finally, very few investigations have examined the effect of ST on specific populations of runners such as young [78], female [32, 38, 72], and masters’ age [86] competitors, therefore future research should attempt to address this dearth in literature.
